# Smooth Muscle Tension Induces Invasive Remodeling of the Zebrafish Intestine

**DOI:** 10.1371/journal.pbio.1001386

**Published:** 2012-09-04

**Authors:** Christoph Seiler, Gangarao Davuluri, Joshua Abrams, Fitzroy J. Byfield, Paul A. Janmey, Michael Pack

**Affiliations:** 1 Department of Medicine, University of Pennsylvania School of Medicine, Philadelphia, Pennsylvania, United States of America; 2 Department of Physiology, Institute for Medicine and Engineering, University of Pennsylvania, Philadelphia, Pennsylvania, United States of America; 3 Department of Cell and Developmental Biology, University of Pennsylvania School of Medicine, Philadelphia, Pennsylvania, United States of America; King's College London, United Kingdom

## Abstract

The signals that initiate cell invasion are not well understood, but there is increasing evidence that extracellular physical signals play an important role. Here we show that epithelial cell invasion in the intestine of zebrafish *meltdown* (*mlt*) mutants arises in response to unregulated contractile tone in the surrounding smooth muscle cell layer. Physical signaling in *mlt* drives formation of membrane protrusions within the epithelium that resemble invadopodia, matrix-degrading protrusions present in invasive cancer cells. Knockdown of Tks5, a Src substrate that is required for invadopodia formation in mammalian cells blocked formation of the protrusions and rescued invasion in *mlt*. Activation of Src-signaling induced invadopodia-like protrusions in wild type epithelial cells, however the cells did not migrate into the tissue stroma, thus indicating that the protrusions were required but not sufficient for invasion in this in vivo model. Transcriptional profiling experiments showed that genes responsive to reactive oxygen species (ROS) were upregulated in *mlt* larvae. ROS generators induced invadopodia-like protrusions and invasion in heterozygous *mlt* larvae but had no effect in wild type larvae. Co-activation of oncogenic Ras and Wnt signaling enhanced the responsiveness of *mlt* heterozygotes to the ROS generators. These findings present the first direct evidence that invadopodia play a role in tissue cell invasion in vivo. In addition, they identify an inducible physical signaling pathway sensitive to redox and oncogenic signaling that can drive this process.

## Abstract 2

Genetic analyses in zebrafish identify a novel physical signaling mechanism that drives formation of invadopodia-like structures and promotes cell invasion in vivo.

## Author Summary

The epithelial cells lining the digestive tract are separated from the connective tissue stroma by a thin layer of extracellular matrix called the basement membrane. During cell invasion, as occurs during cancer metastasis, epithelial cells breach the basement membrane and invade the tissue stroma. The proteases used by invasive cells to degrade basement membrane in vitro are localized in specialized plasma membrane protrusions known as invadopodia. It is not known, however, whether invadopodia are required for cell invasion in vivo or what triggers their formation. Here, we show that epithelial cells in the intestine of the zebrafish mutant *meltdown* form invadopodia-like protrusions and invade the tissue stroma in response to unregulated contractile tone in the surrounding smooth muscle layer. The invadopodia-like protrusions that form in response to this physical signal are required for epithelial cell invasion in this in vivo model, and they can be induced when unregulated smooth muscle contraction is induced by oxidative stress. These findings provide the first direct evidence that invadopodia play a role in tissue cell invasion in vivo and identify a novel inducible physical signaling mechanism that can drive this process.

## Introduction

Physical signaling mechanisms are increasingly recognized as playing an important role in regulating the growth, differentiation, and morphology of vertebrate tissues [1],[2]. In vitro studies have shown that the polarization, shape, and three-dimensional arrangement of cells in culture can be altered by changing the mechanical properties of their underlying substrate [3],[4]. Tissue remodeling in vivo can also be initiated by physical signals. In the vasculature, forces arising from changes in intraluminal pressure can activate membrane-bound ion channels or signaling molecules within endothelial cells [5],[6]. This leads to changes in the architecture of endothelial cells themselves, as well as the surrounding smooth muscle and adventitial cells in the vessel wall. Physical signaling has also been shown to be important in tumor progression. Matrix stiffening promotes tumor cell invasion in breast cancer models and neoplastic transformation of benign papillomas to skin cancers [7],[8]. Mechanical strain induces an oncogene expression profile in intestinal explants derived from tumor prone mice [9]. Cell proliferation within a tumor can alter vascular permeability. This increases interstitial pressure within the tumor itself [10],[11], which promotes tumor progression in animal models [12].

Invasion of cancer cells through their basement membrane is an early event during tumor progression and is a histological feature that distinguishes cancers from benign tumors. In vitro models suggest cancer cell invasion requires the formation of invadopodia, actin-rich membrane protrusions that provide a localized source of matrix degrading proteases [13]–[14]. Invadopodia and structurally related podosomes were first discovered in cells transformed with the Rous Sarcoma Virus oncogene *v-SRC* (reviewed in [14]). High levels of endogenous SRC are thought to promote invadopodia that form spontaneously in invasive cancer cells or following activation of growth factor signaling pathways [14]. Changes in substrate rigidity were recently shown to alter the number and activity of invadopodia that form spontaneously in invasive breast cancer cells [15],[16], thus providing a potential mechanistic link between invasion and physical signaling. Although invadopodia have been studied extensively in cell culture models, their precise role in cell invasion in vivo has not yet been determined [14].

In previous work, we showed that cell invasion can be modeled in a zebrafish mutant, *meltdown* (*mlt*), in which intestinal architecture is disrupted by a mutation in *myosin heavy chain 11* (*myh11*), the gene encoding the principle myosin present in smooth muscle [17]. Biochemical and in vitro analyses showed that the mutant myosin had non-regulated ATPase activity but lacked motor activity. Metalloproteases linked to cancer invasion (Mmp14 (MT1-Mmp); Mmp2) and regulators of epithelial mesenchymal transition were upregulated in *mlt*, and their inhibition rescued the mutant phenotype, thus linking *mlt* to established models of cell invasion. Here we present in vivo evidence that the *mlt* mutation transforms Myh11 into a constitutively active contractile protein, and that unregulated actomyosin interactions increase the basal level of smooth muscle contractile tone in the larval intestine. The physical signal arising from increased contractile tone activates a feed forward redox signaling loop that induces invadopodia-like protrusions in the epithelium, and we show that this requires Tks5, a Src substrate that is a component of the ROS generating Nox complex and is required for formation of invadopodia and podosomes in mammalian cells [18]–[20]. Heterozygous *mlt* mutants normally have no detectable phenotype, however treatment with ROS generators induced both the protrusions and cell invasion. Together, these findings identify a novel inducible in vivo signaling mechanism that non-cell autonomously drives formation of invadopodia-like protrusions and cell invasion.

## Results

### Unregulated Myh11 in *mlt* Drives Formation of Invadopodia-Like Protrusions That Are Required for Invasive Tissue Remodeling

**Figure 1:**
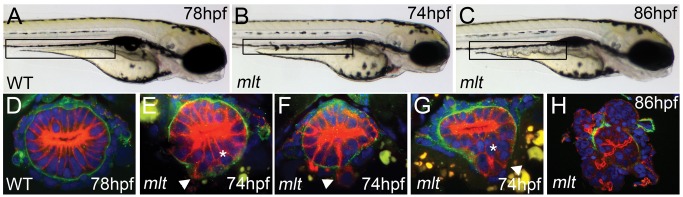
Intestinal epithelial invasion in *mlt* larvae. (A–C) Live images of wild type (WT) and *mlt* larvae. In WT (A) the posterior intestine forms a smooth cylindrical tube (box), whereas in *mlt* at 74 hpf the intestinal contour is irregular (B). Cystic expansion of the intestine is evident in 86 hpf *mlt* larvae (C). (D–H) Histological cross-sections through the posterior intestine of larvae immunostained for laminin (green) and cytokeratin (red). The WT intestine is comprised of a simple epithelial sheet consisting of a single layer of cells, whereas in *mlt* epithelial stratification (asterisks) and invasive cells that have breached the basement membrane are evident (E–G arrowheads). The initial invasive behavior is followed by expansive growth and loss of epithelial architecture (H).

The *mlt* invasive phenotype is first visible at 74 h post-fertilization (hpf), shortly after the onset of *myh11* expression in the developing intestinal circular smooth muscle layer surrounding the epithelium [17]. At this stage, the wild type intestinal epithelium is comprised of a single layer of cells that have formed an apical brush border (Figure 1A and 1D) and are joined to one another by apical tight junctions, adherens junctions, and desmosomes. The cells have a progenitor phenotype as they are still in a phase of proliferative growth and do not express lineage-specific markers [21]. In *mlt*, the intestinal tube has an irregular contour in comparison to the wild type intestine (Figure 1B and 1A). Histological analyses at this stage showed focal regions of basement membrane degradation with invasion of epithelial cells into the surrounding tissue or epithelial stratification (collectively referred to as invasive remodeling) (Figure 1E–1G; *n* = 15 *mlt* and 15 wild type larvae; see also [17]). This initial phase of tissue remodeling is followed by expansive growth of the epithelium and the formation of fluid filled cysts (Figure 1C and 1H).

**Figure 2:**
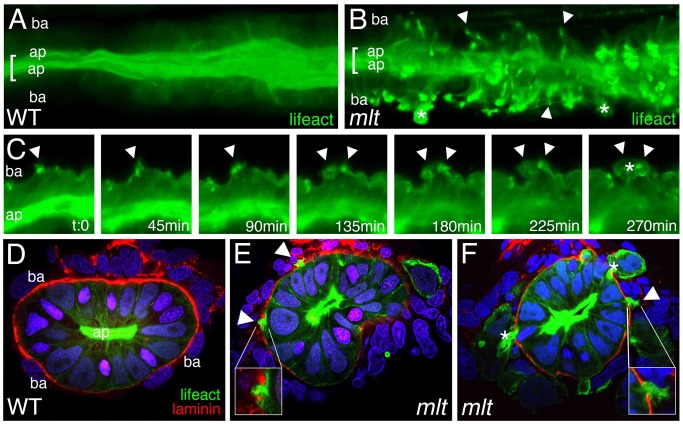
Actin-rich protrusions in invasive epithelial cells of the *mlt* **intestine.** (A, B) Full thickness 3-D rendering of sagittal confocal sections through the intestine of 78 hpf wild type (WT) (A) and *mlt* (B) larvae. Actin is labeled by transgenic Lifeact-GFP expression (green). (A) In WT, the majority of the label is present in the epithelial cell apical brush border (bracket). (B) In *mlt*, actin-rich invadopodia-like protrusions of the basal epithelial cell membrane are detected (arrows), in addition to brush border actin (bracket). (C) Time lapse analysis of protrusion development. Single sagittal confocal scans through the intestine of a *mlt* larva beginning at 74 hpf. Basal invadopodia-like protrusions (arrowheads) precede cell invasion, which is first detected at 135 min. Asterisks mark invasive cells at 270 min (see also B). (D–F) Histological cross-sections through the intestine of 74 hpf immunostained *mlt* larvae. Basement membrane is labeled red (laminin immunostain) and actin labeled green (GFP immunostain in Lifeact-GFP transgenics). Nuclei stained blue with DAPI. Actin rich protrusions in *mlt* co-localize with sites of basal lamina degradation (arrowheads and insets E, F). During progression of the phenotype epithelial cells invade the tissue stroma through degraded regions of the basal lamina (asterisks in F). ap, apical epithelial cell border; ba, basal cell epithelial cell border.

**Figure 3:**
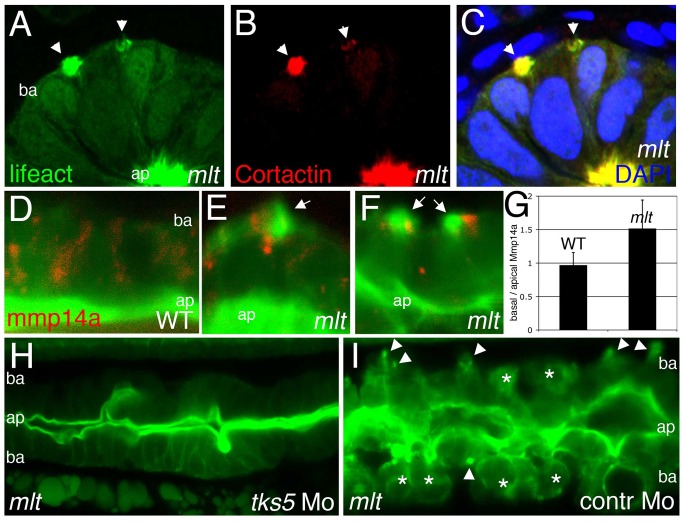
Actin rich protrusions in *mlt* are invadopodia homologs. (A–C) Histological cross-sections through the posterior intestine of 74 hpf *mlt* mutant larvae immunostained with antibodies against GFP (green) (labeling Lifeact-GFP) and Cortactin (red). DAPI-stained nuclei, blue. Lifeact-GFP and Cortactin co-localize in actin rich basal membrane protrusions (arrowheads) and in the apical brush border (ap). (D–G) Sagittal confocal scans through the intestine of 74 hpf Lifeact-GFP transgenic wild type (WT) and *mlt* larvae. Lifeact-GFP binds actin in the apical brush border (ap) of WT (D) and *mlt* (E, F) epithelial cells, as well as basal (ba) invadopodia-like protrusions in *mlt* (E, F, arrows). In WT, the Mmp14a-mCherry fusion protein (red) is distributed throughout the epithelial cells. In *mlt*, Mmp14a-mCherry is preferentially localized to the basal region of the epithelial cells. (*n* = 33 WT and 33 *mlt* cells examined; 6 larvae each genotype). (G) Ratio of basal to apical Mmp14a-mCherry in WT versus *mlt* epithelial cells (error bars, standard deviation. * *p*<.001). (H–I) Sagittal confocal scans through the intestines of 84 hpf Lifeact-GFP transgenic *mlt* larvae. Invadopodia (arrowheads I) and invasive cells (asterisks I) are present in the *mlt* larvae injected with a control morpholino (I), but are not detected in the larvae injected with the *tks5* morpholino (H).

In tissue culture models, degradation of the basement membrane by invasive cells is driven by metalloproteinases associated with invadopodia, plasma membrane protrusions that are enriched in actin, and the actin binding protein Cortactin [14],[22]. In previous work, we showed that invasion in *mlt* required Mmp-14 (also known as MT1-Mmp), a metalloproteinase associated with invadopodia, as well as Mmp2, a metalloproteinase that is activated by Mmp-14 [17]. To determine whether invadopodia-like protrusions were present in the *mlt* intestinal epithelium, we derived a zebrafish transgenic line, *Tg(miR194:Lifeact-GFP)*, in which a GFP-labeled peptide that binds F-actin (Lifeact-GFP, [23]) is expressed in the intestine. In vivo imaging showed that *mlt* epithelial cells form actin rich protrusions at their basal membrane at the stage when matrix degradation and invasion are first detected (Figure 2A and 2B; *n* = 12 *mlt* and 10 wild type larvae; Movies S1 and S2). Time-lapse imaging showed that the protrusions formed before the onset of epithelial cell invasion (Figure 2C; Movies S3, S4, S5, S6) and that they persisted for several hours, thus distinguishing them from other actin-rich protrusions, such as lamellipodia and filopodia [14]. Cross-sections of larvae immunostained with antibodies against laminin and GFP showed that the protrusions localize to sites of extracellular matrix degradation and that epithelial cells invade the surrounding stroma through segments of degraded basement membrane (Figure 2D–F; *n* = 20 *mlt* and 20 wild type larvae analyzed). Immunostainings confirmed that Cortactin, a protein present within invadopodia and that is required for their formation, was present in the actin-rich *mlt* protrusions (Figure 3A–3C; *n* = 18 of 19 cells with protrusions). These findings argue that the protrusions present in the *mlt* intestinal epithelial cells are invadopodia homologs.

MMP-14 localizes to invadopodia, where it drives degradation of the extracellular matrix. To test whether Mmp-14 was present in the *mlt* protrusions we compared the cellular localization of an ectopically expressed Mmp14a-mCherry fusion protein in *mlt* and wild type intestinal epithelial cells. This approach was necessary because none of the antibodies we tested in immunostainings detected endogenous Mmp-14 protein in the intestine. Confocal analyses showed that the Mmp14a-mCherry fusion protein preferentially localized to the basal portion of *mlt* epithelial cells with protrusions (Figure 3D–F), whereas in wild type larvae, Mmp14a-mCherry was evenly distributed within the epithelial cells. Fluorescence quantification showed a 1.5-fold increase in basal Mmp14a-mCherry in *mlt* (Figure 3G). In many *mlt* cells the Mmp14-mCherry fusion protein was nearly exclusively present within or adjacent to the actin-rich protrusions in the basal cell membrane, whereas this pattern was not detected in wild type cells.

To further characterize the *mlt* membrane protrusions, we next asked whether they could form in mutant larvae that were deficient in the Src substrate Tks5, a scaffolding protein that is required for invadopodia and podosome formation in mammalian cells [18]–[20],[24]. Knockdown of zebrafish Tks5 disrupts migration of developing neural crest cells, however it is not known whether these cells form matrix-degrading protrusions similar to *mlt* [25]. Injection of the Tks5 morpholino into newly fertilized wild type and *mlt* embryos caused severe developmental delay in most embryos, as previously reported [25]. However, in the *mlt* embryos that developed normally Tks5 knockdown blocked formation of the protrusions and rescued the invasive phenotype (Figures 3H, 3I, and S1; *n* = 5 independent experiments; confirmed by genotyping in six rescued *mlt* homozygotes).

Together, these findings argue that the membrane protrusions present in *mlt* epithelial cells are closely related, if not identical to invadopodia and or podosomes, and furthermore, that the protrusions are required for invasion. Because podosomes have not been described in epithelial cells and are associated with cell migration rather than invasion, hereafter we refer to the *mlt* protrusions as invadopodia-like protrusions.

### Constitutively Active *src* Induces Formation of Invadopodia-Like Protrusions and Basement Membrane Degradation but Not Cell Invasion

**Figure 4:**
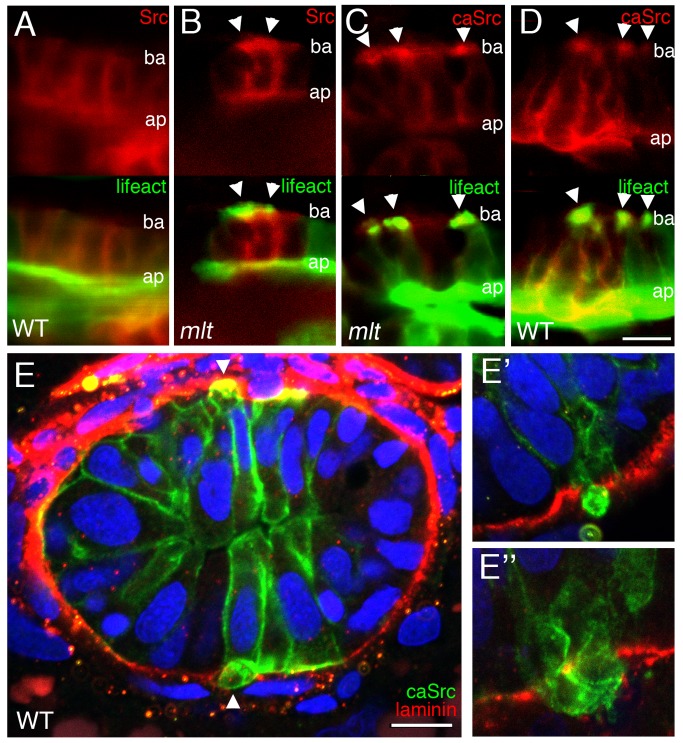
Src induces formation of invadopodia-like protrusions in the intestine of wild type zebrafish larvae. (A–D) Sagittal confocal scans through the intestine of 74 hpf wild type and *mlt* larvae that express Src-mCherry (red) and Lifeact-GFP (green) in the intestinal epithelium. (A) In WT, Src (red) is localized at the apical (ap) and lateral epithelial cell membrane. (B) In *mlt*, Src also localizes to sites of actin-rich (green) invadopodia-like protrusions (arrowheads B) arising from the basal epithelial cell membrane (ba). (C) Constitutively active Src (caSrc; red) localizes to invadopodia-like protrusions (green) in *mlt* (arrowheads). (D) caSrc induces formation of the protrusions in WT (arrowheads). (E) Histological cross-sections through the intestine of a 74 hpf wild type larva showing caSrc-rich protrusions (green) protruding through small degraded regions of the basal lamina (laminin immunostain, red). Additional examples are shown in high power images (E, E′, and E″).

Invadopodia have first been described in cells transformed with the Rous Sarcoma Virus oncogene, *v-src* [26],[27]. SRC is a major organizing protein of invadopodia and is present within invadopodia [28]. To localize Src in *mlt* intestinal epithelial cells, we expressed a fusion construct encoding Src with C-terminal mCherry in the epithelium. Confocal microscopy showed that Src-mCherry was present at the apical and lateral membrane of WT epithelial cells (Figure 4A; *n* = 40 cells examined in eight larvae), but was concentrated at the basal cell membrane of invasive *mlt* epithelial cells that formed invadopodia-like protrusions (Figure 4B; *n* = 25 single cells in five larvae).

To test whether Src was sufficient to induce formation of the invadopodia-like protrusions in the absence of the mutant Myh11, we expressed an activated form of zebrafish Src in which the conserved inhibitory Tyrosine phosphorylation site was replaced with a Phenylalanine (Src-Y528F; hereafter caSrc). This mutant form of Src induces invadopodia formation in non-transformed cells [14]. Similar to wild type Src, a caSrc-mCherry fusion protein localized to basal protrusions in the intestinal epithelium of *mlt* mutant larvae (Figure 4C; *n* = 40 cells in eight larvae). caSrc-mCherry (or caSrc-GFP) induced formation of actin-rich protrusions at the basal surface of the wild type epithelial cells by 78 hpf (Figure 4D). Anti-laminin and anti-GFP immunostaining showed that the Src-induced protrusions were present at sites of basement membrane degradation (Figure 4E–4E″; *n* = 53 cells examined: 40 with basal protrusions, of which 27 breached the basement membrane). However, none of the cells with protrusions were invasive in either 74 hpf larvae, or larvae followed to 5 d post-fertilization. The 5 d post-fertilization (dpf) transgenic larvae also had far fewer protrusions than the 74 hpf larvae (Figure S2), indicating a higher capability of protrusion induction at earlier time points, when the epithelial cells are highly proliferative and not fully differentiated [20].

### Src Is Required for Epithelial Cell Invasion in *mlt*

**Figure 5:**
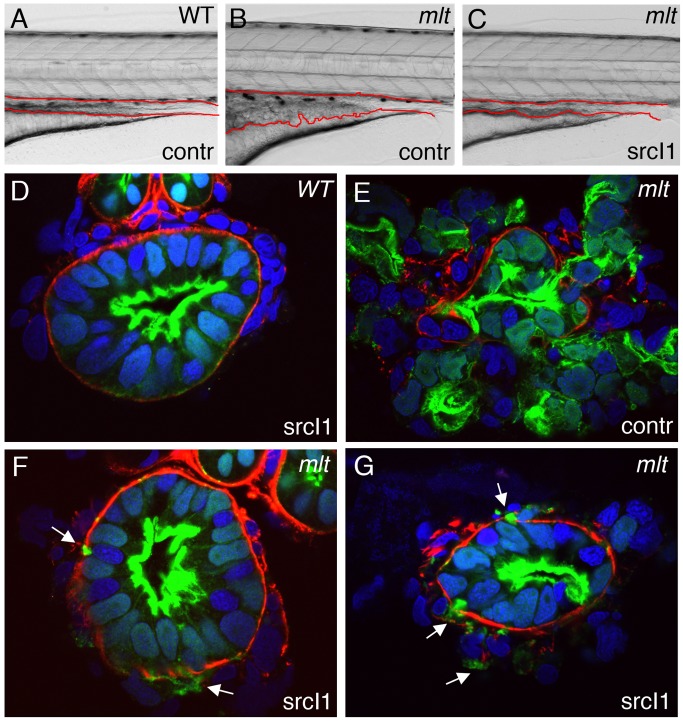
Src inhibition rescues invasion but not formation of invadopodia-like protrusions in *mlt*. (A–C) Lateral views of live 5 dpf WT (A) and *mlt* (B, C) larvae. Treatment with the Src-I1 inhibitor rescues invasion in *mlt* (C). The size of the intestinal epithelium in the treated *mlt* larva (C) is reduced compared with the untreated *mlt* larva (B). (D–G) Histological cross-sections of 4 dpf WT (D) and *mlt* (E–G) *Tg(miR194:Lifeact-GFP)* larvae immunostained with antibodies against laminin (red) and GFP (green). Nuclei stained blue with DAPI. Arrows point to invadopodia-like protrusions arising from the basal epithelial cell membrane of Src-I1 treated *mlt* larvae (F, G). Pronounced invasion with distortion of intestinal architecture is evident in the untreated *mlt* larva (E). Note that invasion is markedly reduced in the Src-I1 treated *mlt* larvae despite the presence of the invadopodia-like protrusions (white arrows in F, G).

We next tested whether Src contributed to invasion in *mlt*. Mutant larvae and larvae expressing the caSrc transgene were treated with three established mammalian Src inhibitors: SU6656, PP2, and Src-I1 (Figure 5) [29]. SU6656 blocks cell migration during gastrulation and neural crest development in zebrafish embryos [25],[30], however it had no effect in *mlt*, nor did it block formation of the invadopodia-like protrusions that form in response to caSrc (*n* = 21 larvae). PP2 blocked formation of the invadopodia-like protrusions induced by caSrc (*n* = 0 protrusions detected in 34 larvae), but it had no effect in *mlt* (*n* = 20 larvae). In contrast, the Src-I1 inhibitor had a pronounced effect on both the formation of the invadopodia-like protrusions in response to caSrc (0 protrusions detected in 33 larvae) and on invasion in *mlt* (*n* = 21 *mlt* larvae). The effect of Src-I1 was comparable to the Tks-5 knockdown (compare Figures 5 and S1). Although Src-I1 treatment had a profound effect on invasion in *mlt*, it did not block formation of the invadopodia-like protrusions (Figure 5F, 5G). The response of *mlt* to Src-I1 is similar to the effect of Src knockdown in invasive breast cancer cells [24]. Actin-rich invadopodia precursors form in the Src-deficient cancer cells, but invasion and to a lesser extent matrix degradation are both inhibited.

### The *mlt* Mutation Causes an Increase in the Basal Level of Intestinal Smooth Muscle Contractile Tone

**Figure 6:**
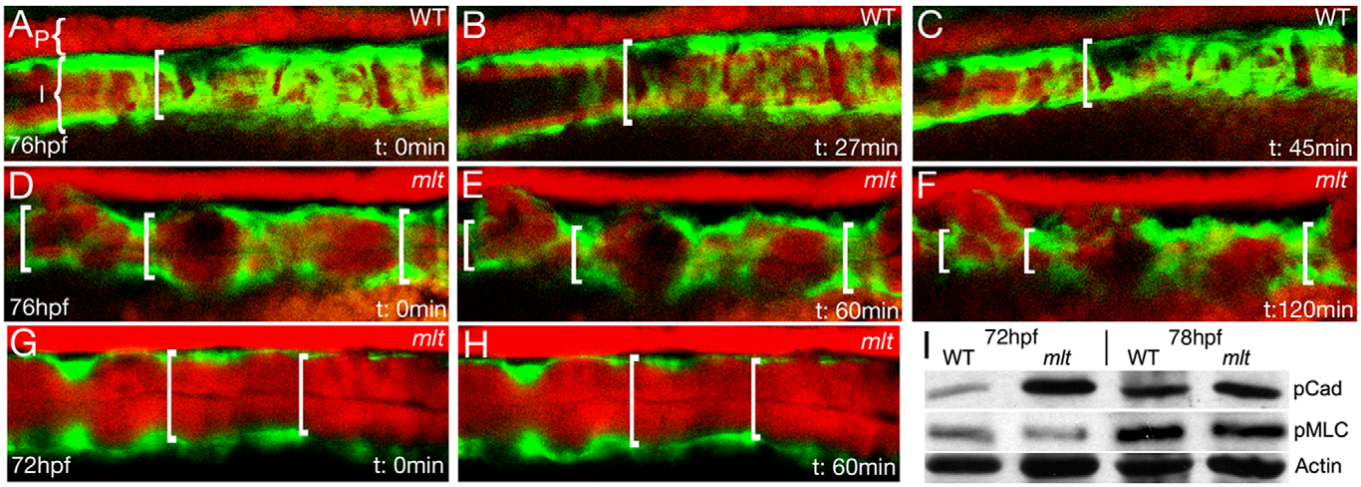
Tonic intestinal smooth muscle contraction in *mlt* mutants. Sagittal confocal images from time lapse movies (Movies S7 and S8) of transgenic WT (A–C) and *mlt* (D–H) larvae (anterior left) expressing fluorescent reporters. Intestinal epithelium (I) and pronephric duct (P) labeled red; intestinal smooth muscle labeled green. WT images beginning at 76 hpf show cycling of the contracted (A, C) and relaxed state (B); brackets depict intestinal diameter. (D–F) The *mlt* intestine at this stage shows progressive contraction that disrupts intestinal architecture. (G, H) Smooth muscle contraction is not detected in the *mlt* intestine at the onset of invasive remodeling (72 hpf). (I) Western blot showing premature phosphorylation of h-CaD (p-Cad) in 72 hpf *mlt* larvae, before the onset of smooth muscle contraction (ratio WT:*mlt* = 0.17/1, relative to Actin loading control; in four independent experiments, the ratio averaged 0.13/1; *p* = 0.002). Phospho-h-CaD is present at the onset of contraction in WT at 78 hpf and is comparable to *mlt* (ratio WT:*mlt* = 1.12/1, relative to Actin; no significant difference in four independent experiments). Levels of phospho-Myosin light chain (p-Mlc) are low at 72 hpf in both *mlt* and WT (ratio WT:*mlt* = 1.16/1, relative to Actin) and are increased when contractions occur at 78 hpf (ratio WT:*mlt* = 0.96/1, relative to Actin). There was no significant difference between WT and *mlt* at either time point in four independent experiments. Actin (beta-Actin), loading control.

Mechanical signaling modulates the formation and activity of invadopodia [15],[16]. This suggests the *mlt* protrusions formed in response to mechanical cues from the unregulated mutant Myh11. To test this idea, we derived transgenic *mlt* larvae expressing fluorescent reporters in smooth muscle [31] and the intestinal epithelium (*Tg(miR194:mCherry; sm22a:GFP)*) and recorded intestinal peristalsis using time-lapse confocal microscopy. Contractile force in smooth muscle arises from the interaction of Smooth muscle actin (Sma) and myosin (Myh11) filaments that are anchored to the cortical cytoskeleton [32]. In zebrafish larvae, intestinal smooth muscle contraction begins around 76 hpf [33], several hours after Sma and Myh11 are first detected. We detected rhythmic peristaltic contractions of the circular smooth muscle layer surrounding the epithelium in wild type larvae at this stage (Figure 6A–6C and Movie S7; *n* = 6 larvae examined). By contrast, slow tonic smooth muscle contraction that distorted tissue architecture was evident in 76 hpf *mlt* mutants (Figure 6D–6F and Movie S8; *n* = 6 larvae examined). These findings indicated that the mutant myosin had non-regulated contractile activity that was not detected in our original in vitro motility assays [17], presumably because of the slow rate of contraction.

Invasive cells are already present in *mlt* larvae at 72 hpf (Figure 1E and 1F), however at this stage we could not detect tonic or peristaltic smooth muscle contraction in either mutant or wild type intestines (Movies S9 and S10; Figure 6G and 6H; *n* = 6 *mlt* and 6 wild type larvae), as previously reported [33]. This led us to hypothesize that the mutant myosin increased the resting or basal level of smooth muscle contractile tone in the intestine, and that increased tone triggered formation of the invadopodia-like protrusions.

To further characterize smooth muscle contractility in *mlt* we examined levels of smooth muscle regulatory proteins in the mutant and wild type intestine using Western blot analyses. The enteric nervous system regulates intestinal peristalsis by controlling levels of the phosphorylated form of the smooth muscle regulatory Myosin light chain (p-Mlc) that binds Myh11 [34],[35]. Mlc phosphorylation activates the Myh11 ATPase, which in turn drives crossbridge cycling of the actomyosin complex (and hence contraction). We have previously identified an antibody that detected a p-Mlc isoform whose expression is restricted to the smooth muscle layer of the larval intestine [34]. Western blot analyses using this antibody showed that p-Mlc levels were low in wild type larvae before the onset of peristalsis (72 hpf) and were significantly higher 6 h later (78 hpf), when circular smooth muscle contractions are evident (Figure 6I). p-Mlc levels in *mlt* were similar to wild type at both time points (Figure 6I). These findings are consistent with previous work showing that the ATPase activity of the mutant Myh11 is independent of Mlc phosphorylation (i.e., unregulated) [17].

We next examined levels of the high molecular weight isoform of Caldesmon (h-CaD), a smooth muscle–specific actin binding protein that regulates contractile tone independently of p-Mlc [36]–[39]. h-CaD is expressed exclusively in the smooth muscle of the zebrafish larval intestine, where it binds Myh11and Sma, but not Actb (beta-Actin), the predominant actin in the cytoskeleton [34],[39]. In its non-phosphorylated state, h-CaD inhibits Sma-Myh11 interactions, most likely by acting as a brake on actomyosin crossbridges [38]. When h-CaD is phosphorylated, its interactions with Sma are weakened. This enhances contractile force. Western blots showed that h-CaD was prematurely phosphorylated in the *mlt* intestine before the onset of active peristaltic contraction (72 hpf), when invasion is first detected (Figure 6I; *n* = 6 independent experiments). Premature h-CaD phosphorylation in *mlt* is consistent with the idea that resting smooth muscle tone is increased in the intestine of the mutant larvae.

### Modulation of Smooth Muscle Tone Can Induce or Suppress Invasive Remodeling in *mlt*

**Figure 7:**
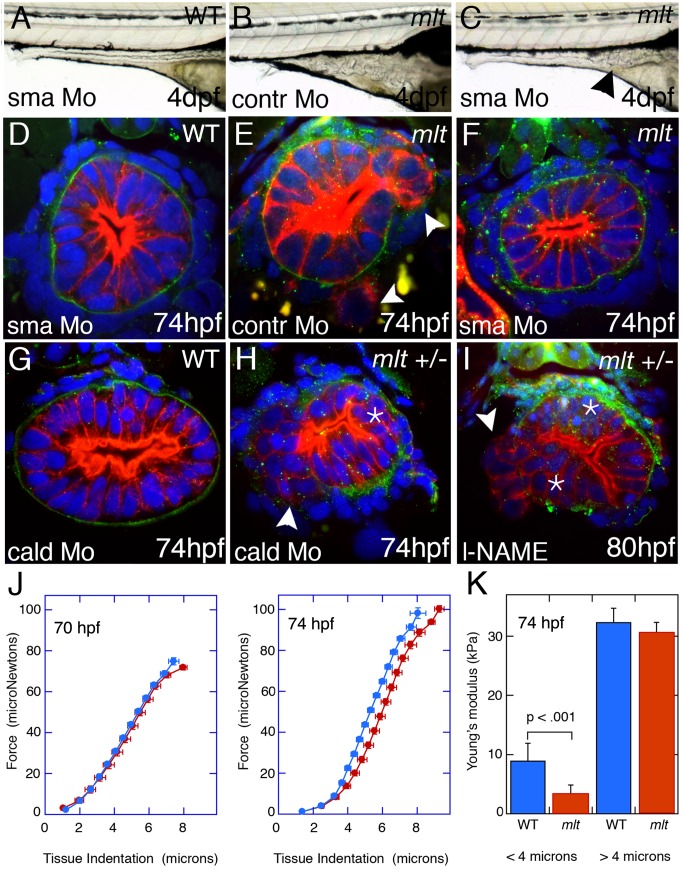
Smooth muscle contraction drives epithelial invasion but does not alter tissue rigidity. (A–C) Lateral views of live 5 dpf larvae injected with Sma or control morpholino. (A) Sma knockdown has no effect on WT intestinal morphology. (B) Control morpholino injection in *mlt*. (C) Sma knockdown rescues invasion in *mlt*. Residual invasive cells persist in this Sma deficient *mlt* larva (arrowhead). (D–I) Histological cross-sections through the posterior intestine of 74 hpf WT and *mlt* larvae immunostained with anti-keratin (red) and anti-laminin (green) antibodies. (D) WT. (E, F) *mlt* larvae injected with control (E) and Sma (D, F) morpholinos. Invasive cells in *mlt* (arrowheads, E) are rescued by Sma knockdown. (G, H) Injection of a morpholino targeting the high molecular weight isoform of Caldesmon (cald) has no effect on WT intestinal morphology but induces invasion (arrowhead) and stratification (asterisk) in an *mlt* heterozygote. (I) Treatment of an *mlt* heterozygote with L-NAME causes invasion (arrowhead) and epithelial stratification (asterisk). (J) Force displacement measurements show identical compliance of intestines dissected from *mlt* and WT larvae before the phenotype develops at 70 hpf, and a modest increase in compliance at the outer surface of the intestine (<4 micron indentation) when invasion is present at 74 hpf. (K) Compliance is indicated by Young's modulus, which is proportional to the slope of the Force versus Tissue indentation curve.

To directly examine whether smooth muscle contractile tone initiated epithelial invasion in *mlt* we inhibited translation of the mRNA encoding zebrafish Sma [34],[40]. The morpholino used to target Sma does not target Actb, the cytoskeletal actin isoform that also is present in smooth muscle but does not interact with Myh11 [32],[34]. Knockdown of Sma rescued the early *mlt* invasive phenotype at 74 hpf (Figure 7A–7F; no invasion in 120 larvae from a *mlt/+* intercross; confirmed histologically in 5 *mlt* homozygotes) as well as epithelial expansion and cysts that occur at later stages. Western blots confirmed the efficacy of the Sma knockdown and that it did not alter levels of Myh11 (Figure S3). Together, these findings show that smooth muscle contraction is required for the mutant phenotype and that the constitutive ATPase activity of the mutant Myh11, which is independent of the presence of Sma [17], is not sufficient to trigger invasion.

We next asked whether enhancing contractile tone was sufficient to induce invasion in heterozygous *mlt* mutants. To test this idea, we inhibited translation of the h-CaD mRNA using a splice blocking morpholino that specifically targets this smooth muscle–specific CaD isoform [38]. We chose this approach because levels of phospho-h-CaD in heterozygous larvae are low at the stage when invasion is first detected in *mlt* homozygotes (72 hpf; Figure 6I). h-CaD knockdown was therefore predicted to increase smooth muscle contractile tone (as reported in vascular smooth muscle [38],[41]), similar to the effect of premature h-CaD phosphorylation in homozygous mutants. Indeed, h-CaD knockdown caused epithelial invasion and stratification in *mlt* heterozygotes, but had no effect in wild type larvae besides increasing the rate of intestinal transit (Figures 7G, 7H, and S4; invasion detected in 69% larvae from a *mlt/+* intercross (*n* = 240) of which 66% were predicted to be *mlt/+*; 12 of 12 genotyped larvae were *mlt/+*) [39]. A similar epithelial response was seen in the heterozygotes treated with L-NAME, a nitric oxide synthase (Nos) inhibitor that increases intestinal smooth muscle contraction in zebrafish larvae (Figure 7I; invasion was histologically confirmed in 11 genotyped *mlt/+* larvae) [42]. Unlike homozygous *mlt* mutants, neither h-CaD-deficient nor L-NAME-treated heterozygotes developed epithelial cysts or other features of the advanced homozygous *mlt* phenotype, thus indicating that these phenotypic features require sustained smooth muscle contraction.

### Distinct Mechanosensory Mechanisms Are Activated in Invasive Cells by Smooth Muscle Tension and Matrix Stiffening

Matrix stiffening promotes cell invasion in breast cancer models, in part through activation of Focal Adhesion Kinase (FAK) [3],[7]. To determine whether a comparable mechanotransductive signaling mechanism was activated in *mlt* we measured tissue elasticity in intestines isolated from wild type and mutant larvae using a force displacement assay [43]. Before invasive remodeling is detected (70 hpf), compliance was nearly identical in *mlt* larvae and their wild type siblings throughout the range of indentations tested (Figure 7J; *n* = 6 *mlt* and 21 wild type larvae). When remodeling is first detected (74 hpf), compliance was slightly increased at the surface of the intestine but comparable at greater depths (Figure 7J,K; *n* = 7 *mlt* and 12 wild type larvae examined). Values compatible with intestinal stiffening (reduced compliance) were never recorded.

We next examined Fak phosphorylation in *mlt* using immunostainings [44] and Western blot analyses. Neither detected elevated p-Fak in *mlt* (Figure S5, unpublished data). Western blots also showed that levels of Collagen-1 and Fibronectin were not significantly elevated in the *mlt* intestine (Figure S5). All together, these data argue that mechanical force triggers epithelial invasion in *mlt* independently of changes in matrix composition or increases in tissue rigidity.

### Smooth Muscle Tension Activates Epithelial Redox Signaling in *mlt* Homozygotes and Pharmacologic Activation of Redox Signaling Induces Epithelial Cell Invasion in *mlt* Heterozygotes

**Figure 8:**
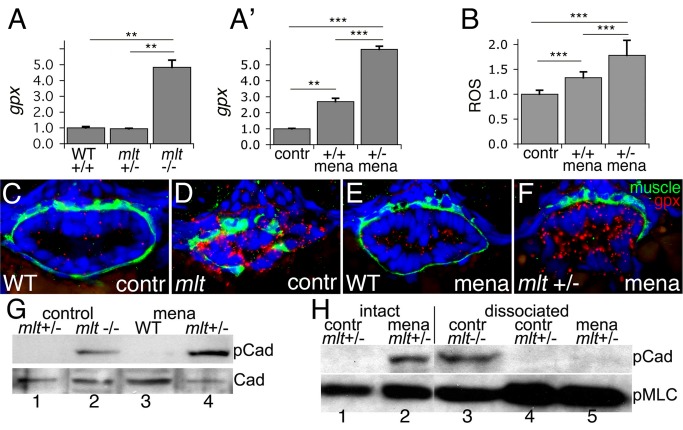
Heterozygous *mlt* larvae are sensitized to oxidative stress. (A) Quantitative RT-PCR shows increased intestinal *gpx* expression in 74 hpf *mlt* homozygotes (*mlt* −/−) compared with WT (+/+) and *mlt* heterozygotes (*mlt +/*−) (** *p*<.01). (A′) Quantitative RT-PCR shows intestinal *gpx* expression is increased in menadione treated homozygous WT larvae (+/+ mena) compared with untreated WT (contr). A much stronger response to menadione is seen in *mlt* heterozygotes (+/− mena) (*** *p*<.001). (B) ROS production in intestinal epithelial cells of 76 hpf WT control larvae (contr) versus menadione treated homozygous WT and heterozygous *mlt* larvae (*** *p*<.001). Bar graphs in (A) and (A′) show mean and standard deviation of three independent experiments. Bar graph in (B) shows mean of six larvae for each genotype; 15–25 cells per larva. (C–F) Histological cross-sections of larvae processed for fluorescent RNA in situ hybridization. Menadione induced *gpx* expression (red) in the epithelium but not smooth muscle (green) of heterozygous *mlt* larvae (F). This is comparable to the *gpx* expression pattern in control homozygous *mlt* larvae (D). (G) Western blot showing premature h-CaD phosphorylation (pCad) in dissected intestines from menadione treated heterozygotes but not homozygous WT larvae (lane 4 versus lane 3; ratio phospho-h-CaD WT:*mlt* = 0.02/1, relative to total h-CaD; CaD; in six experiments the ratio averaged 0.016/1; *p*<.001). h-CaD is prematurely phosphorylated in intestines dissected from 74 hpf *mlt* homozygotes versus *mlt* heterozygotes (lane 1 versus lane 2). (H) Western blot showing h-CaD phosphorylation (pCad) in the menadione treated heterozygous intestines prior to dissociation (lane 2) but not after dissociation into free cell populations (lane 5). Phospho-h-CaD persists in dissociated cells from homozygous intestines (lane 3), but is not detected in control intestines dissected from *mlt* heterozygotes, before (lane 1) or after (lane 4) dissociation into free cell populations. No phopho-h-CaD was detected in any dissociated samples in three independent experiments. Loading control, phospho-Myosin light chain (pMlc).

To identify signaling pathways activated by increased smooth muscle tension in *mlt* we performed microarray transcriptional profiling of early (74 hpf) mutants. Among the group of upregulated genes were three that neutralize reactive oxygen species (ROS) (*glutathione peroxidase* (*gpx*), *thioredoxin*, and *glutathione/thioredoxin reductase*) and several members of the AP-1 family of transcription factors (Table S1), which are also ROS-responsive genes. Genes encoding MAP kinase regulators were also activated in *mlt*. The microarray findings were confirmed by quantitative RT-PCR experiments (Figures 8A and S6; *n* = 15 intestines per genotyped pool; three pools examined), RNA in situ hybridization (Figures 8C, 8D, and S6), and Western blot analyses (Figure S6). Fluorescent RNA in situ hybridization experiments showed that *gpx* and the AP-1 transcription factor *junB* were nearly exclusively expressed within the *mlt* intestinal epithelium (Figures 8C, 8D, and S6; *n* = 10 *mlt* and 10 wild type larvae), thus arguing that their expression is non-cell-autonomously activated in response to smooth muscle tension. These findings are noteworthy because there is extensive evidence linking ROS and AP-1 factors to cancer cell invasion through their regulation of expression and activation of metalloproteinases [45]–[47], EMT regulators [48], and the induction of invadopodia [49]–[51].

To test if redox signaling was required to induce invasive epithelial remodeling in *mlt* we treated 72 hpf larvae with the ROS quenchers, N-acetylcysteine, and Tiron. This treatment did not rescue the *mlt* invasive phenotype, but it also did not affect epithelial *gpx* expression (unpublished data), suggesting that the neutralizing compounds could not access the ROS or that the inhibition was not strong enough to inhibit redox signaling.

**Figure 9:**
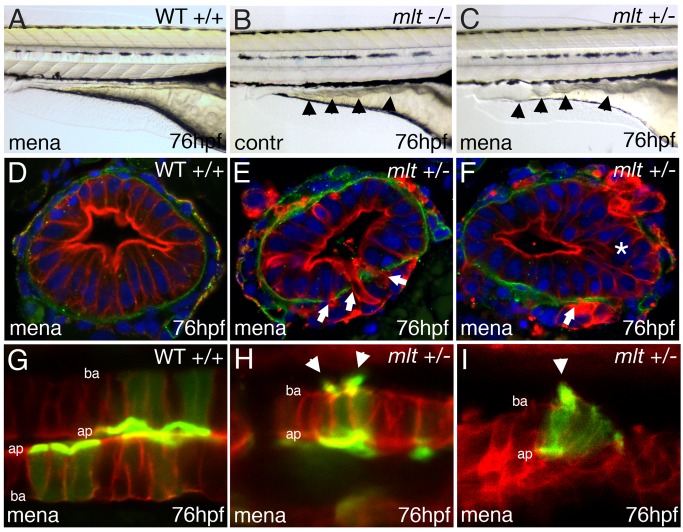
Oxidative stress induces invasive remodeling in *mlt* heterozygous larvae. (A–C) Lateral images of live WT (A), *mlt* homozygous (B), and *mlt* heterozygous larvae (C). The WT and *mlt* heterozygous larvae received 3 h of treatment with Menadione beginning at 73 hpf. Menadione treated heterozygote (C) larvae have an intestinal phenotype (arrowheads) resembling the untreated *mlt* homozygous larvae (B). (D–F) Corresponding histological cross-sections (representative of larvae in A, C) with intestinal epithelial cells labeled red (anti-keratin immunostain) and basement membrane in green (anti-laminin immunostain). Menadione causes epithelial cell invasion (arrows) and stratification (asterisks) in *mlt* heterozygous larvae (E, F) but does not affect epithelial architecture in the WT intestines (D). (G–I) Sagittal confocal scans through the intestine of WT and *mlt* heterozygotes treated with menadione. Both larvae express LifeAct-GFP in a subset of intestinal epithelial cells. Actin-rich invadopodia-like protrusions (green) are seen arising from the basal epithelial cell membrane of menadione treated heterozygous larvae (arrowheads, H, I). Actin is located nearly exclusively in the apical brush border of WT epithelial cells (G): Red -membrane mCherry; ba, basal epithelial cell border; ap, apical epithelial cell border.

To determine whether oxidative stress was sufficient to induce the formation of the invasive phenotype, we treated 72 hpf larvae with intracellular ROS generators menadione and LY83583 [52],[53]. Neither compound generated a *mlt* phenocopy in homozygous wild type larvae and both were lethal during prolonged exposure. However, within 3 h of exposure both compounds induced epithelial invasion in 72 hpf *mlt* heterozygotes that was comparable to early homozygous mutants (Figure 9A–9F). Invasive morphology present in 90% of genotyped *mlt/+* larvae (*n* = 22) but absent from all +/+ wild types (*n* = 17). Invasion was histologically confirmed in all *mlt* (*n* = 12) larvae examined but was not present in wild types (*n* = 9). The treated heterozygotes did not develop the cysts and epithelial expansion typical of the late *mlt* phenotype (unpublished data). Together, these findings suggest that redox signaling is involved in the initial remodeling of the mutant intestine, but on its own it is not sufficient to cause the more pronounced architectural changes seen in more advanced homozygous mutants.

Having established a role for redox signaling in *mlt* we next asked whether menadione induced formation of invadopodia-like protrusions in the heterozygous epithelium. Live confocal imaging of menadione-treated heterozygous larvae showed protrusions that were identical to those present in homozygous larvae (Figure 9H and 9I; *n* = 10 larvae). By contrast, the protrusions were not seen in menadione-treated wild type larvae (Figure 9G; *n* = 10 larvae) or heterozygous larvae that did not receive menadione (*n* = 10 larvae). Interestingly, caSrc not only induced invadopodia-like protrusions in wild type epithelial cells, but also activated expression of the redox responsive gene *gpx*, as occurs in *mlt* (Figure S7; *n* = 12 larvae). These findings establish further links between Src, redox signaling, and formation of the invadopodia-like protrusions in *mlt*.

### Smooth Muscle Tension Activates a Feed Forward Signaling Loop That Amplifies Epithelial Redox Signaling

One explanation that could account for the invasive response of *mlt* heterozygotes to menadione was that activation of redox signaling increased smooth muscle contractile tone, similar to the effect of h-CaD knockdown or Nos inhibition (Figure 7D–7F). Supporting this hypothesis, Western blots showed that menadione induced premature phosphorylation of h-CaD in *mlt* heterozygotes, but not in sibling larvae that were homozygous for the wild type *myh11* allele (Figure 8G). To determine whether h-CaD phosphorylation occurred downstream of smooth muscle redox signaling, we used fluorescent RNA in situ hybridization to localize *gpx* and *junB* expression in the menadione-treated larvae. Expression of both genes was restricted to the epithelium (Figure 8E and 8F; *n* = 10 larvae), as in *mlt* homozygotes (Figures 8C,D, S6). Together, these findings led us to hypothesize that h-CaD phosphorylation in the menadione-treated heterozygotes was triggered by an epithelial signal rather than a direct effect of menadione on the smooth muscle.

To test this hypothesis, we asked whether menadione induced h-CaD phosphorylation in dissociated heterozygous smooth muscle cells. Intestines were dissected from heterozygous *mlt* and wild type larvae and divided into two groups. The first group was treated with menadione and then processed for Western blot analyses. The second group was dissociated into a suspension of epithelial and smooth muscle cells prior to treatment with menadione. We reasoned that the dissociated smooth muscle cells could still directly respond to mendaione even though their interactions with the epithelium and extracellular matrix had been disrupted. Western blots (Figure 8H) showed that h-CaD was phosphorylated in the non-dissociated heterozygous intestines treated with menadione, and that phospho-h-CaD already present in homozygous *mlt* smooth muscle cells prior to cell dissociation was not degraded during the incubation period of the assay. In contrast, phospho-h-CaD was not detected in the dissociated heterozygous smooth muscle cells exposed to menadione (*n* = 3 independent experiments).

**Figure 10:**
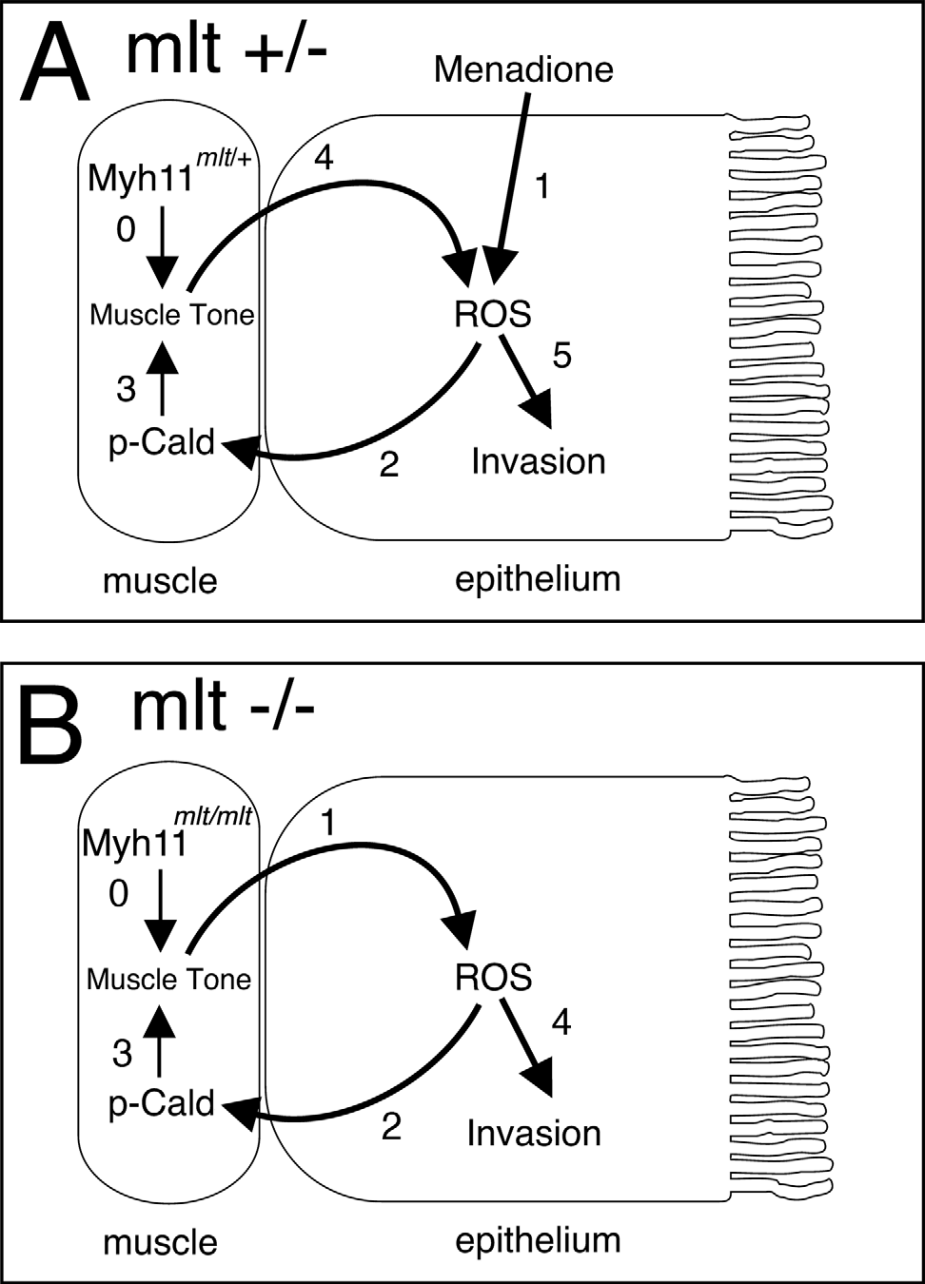
An amplification feedback signaling loop controls smooth muscle contraction and invasion in *mlt* larvae. (A) In *mlt* heterozygotes the expression of mutant Myh11 protein does not generate sufficient smooth muscle tension to induce epithelial invasion or stratification (0). Menadione treatment induces ROS production in the epithelium (1). Epithelial ROS signaling leads to premature phosphorylation of Caldesmon in heterozygous smooth muscle cells (2). The resulting increase in smooth muscle tone (3) leads to an amplified ROS response in the epithelium (4), establishing a feed forward loop causing additional h-CaD phosphorylation and increased smooth muscle tension. Together these stimuli induce invasive remodeling of the epithelium (5). (B) Endogenous smooth muscle tone in *mlt* homozygous larvae (0) induces epithelial ROS (1) and h-CaD phosphorylation via epithelial signaling (2, 3). Ultimately, this culminates in epithelial invasion (4), as in menadione treated *mlt* heterozygotes.

Collectively, these data support a model in which h-CaD phosphorylation in *mlt* smooth muscle cells arises from an ROS-activated epithelial signal (Figure 10). Phosphorylation of h-CaD enhances smooth muscle tone, thereby generating additional oxidative stress within the epithelium. This establishes a feed-forward signaling loop with the adjacent smooth muscle that further enhances contractile tone, amplifies epithelial ROS production, and culminates in epithelial invasion. To test this model we measured epithelial ROS production in menadione-treated wild type and heterozygous larvae that express a ratiometric ROS sensor [54]. Oxidation of the sensor by ROS causes a shift in its fluorescence absorption-emission ratio, thus allowing ROS quantification. Menadione treatment led to a 33% increase in epithelial ROS levels in wild type transgenic larvae (*Tg(beta-Actin:grx-roGFP2*) (Figure 8B). This response was increased more than 2-fold in heterozygous larvae, thus confirming amplification of ROS production in the invasive cells. Quantitative RT-PCR experiments showed that menadione caused a nearly identical increase in intestinal *gpx* and *jun-b* expression in heterozygotes compared with menadione-treated wild type larvae (Figures 8A′ and S6). Indeed, *gpx* and *jun-b* expression in the menadione-treated heterozygotes was comparable to untreated homozygous mutants (Figures 8A and S6). These findings not only support the amplification signaling model, but also confirm that *gpx* and *jun-b* are responsive to ROS and that their expression can be used to measure ROS production.

### Epithelial Oxidative Stress Induces Invasion in Response to Oncogenic Signaling in *mlt* Heterozygotes

**Figure 11:**
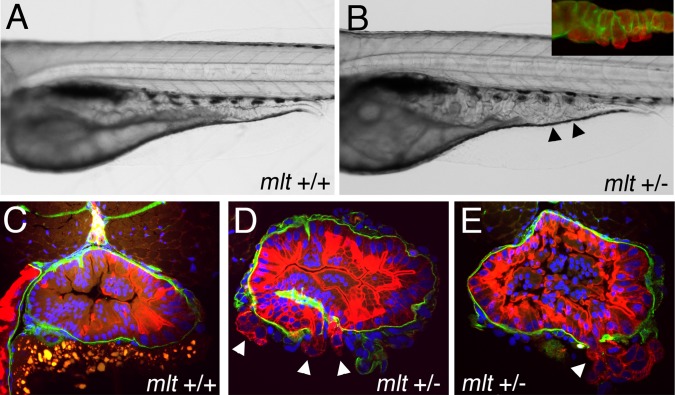
Activation of oncogenic signaling enhances sensitivity of *mlt* heterozygotes to oxidative stress. (A, B) Lateral views of live, menadione treated 5 dpf *axin* mutant larvae that express mutant *KRAS* in the intestinal epithelium (*Kras-axin*). (A) Hypertrophy of the intestinal epithelium in a *Kras-axin* larva that is homozygous for the wildtype *myh11* allele (mlt +/+) larvae is unchanged by treatment with menadione. (B) Menadione treatment causes pronounced cystic expansion of the posterior intestinal epithelium of the *Kras-axin mlt* heterozygote (arrowheads) that resembles the homozygous *mlt* phenotype. Inset, immunolabeling of the basal lamina (laminin, green) and epithelium (cytokeratin, red) shows epithelial cell invasion through the basement membrane. (C–E) Histological cross-sections through the intestine of immunostained larvae show invasive cells in menadione treated *Kras-axin mlt* heterozygotes (arrowheads; D, E). Invasion is not detected in menadione-treated *Kras-axin mlt* larvae that are homozygous for the wildtype *myh11* allele (C).

Heterozygous *mlt* mutants had a pronounced response to menadione when treatment began at 74 hpf, however invasion was not detected when larvae were treated at later stages (5 dpf). We hypothesized that this was caused by a change in the responsiveness of the epithelium to redox signaling. To test whether the responsiveness of the 5 dpf epithelium to menadione was modified in a tumor model, we generated transgenic larvae that express an activated human *KRAS* allele in the intestinal epithelium (*Tg(miR194:eGFP-KRAS^G12V^)*). The *KRAS* transgenics were also homozygous for a loss of function allele of the Wnt regulator *axin1* [55], which like the *KRAS* allele causes intestinal epithelial cell hyperplasia in zebrafish larvae [56],[57]. Previous work in zebrafish has shown that activated KRAS enhances Wnt signaling in Apc-deficient zebrafish larvae and human colorectal tumors [58]. Since zebrafish *apc* mutants have severe developmental delay, the *axin1* mutants were used to study the combined effect of activated *KRAS* and enhanced Wnt signaling in older *mlt* heterozygotes. Prior to treatment with menadione, 5 dpf *axin1* mutants that express the mutant *KRAS* allele (*KRAS-axin* larvae) and were heterozygous for the *mlt* mutation had intestinal epithelial hyperplasia without evidence of invasion. Remarkably, within 5 h of treatment, menadione generated a dramatic invasive response in the *KRAS-axin* larvae that were also heterozygous for *mlt*, whereas it had no effect in larvae that were homozygous for the wild type *myh11* allele (Figure 11; invasion confirmed in 8 of 8 menadione-treated *KRAS-axin* larvae and 0 of 8 untreated *KRAS-axin* larvae).

## Discussion

### Epithelial Invasion in *mlt* Is Triggered by a Tension-Induced Extracellular Physical Signal

In previous work, we reported that invasive remodeling of the developing intestinal epithelium of zebrafish *mlt* mutants is caused by a mutation of the *smooth muscle myosin heavy chain* gene. Initially, we attributed the non-cell-autonomous mutant phenotype to the constitutive actin-independent ATPase activity of the mutant Myh11 protein, rather than its non-regulated contractile function, because contraction was not detected in an in vitro motility assay [17]. Here, we show that the mutant myosin does indeed have contractile function, and that the non-regulated motor activity of the mutant Myh11 is necessary and sufficient for the mutant phenotype. Specifically, we show that inhibiting smooth muscle contraction, via Sma knockdown, rescues invasion in homozygous mutants, and that enhancing smooth muscle contractility, via h-CaD knockdown and Nos inhibition, induces invasion in heterozygous *mlt* mutants. Together, these findings are consistent with the idea that invasive transformation of the *mlt* intestinal epithelium is triggered by a physical signal from the adjacent smooth muscle layer, and that on its own, the unregulated ATPase activity of the mutant myosin (which is independent of actin) is not sufficient to induce invasion.

Physical signaling mechanisms are increasingly recognized as important for organ development and disease [59],[60]. Recent studies suggest a role for changes in matrix rigidity (stiffness) and other types of mechanical forces in cancer progression [3],[7]–[12],[61]. Our findings distinguish *mlt* from these cancer progression models in several ways. First, invasive remodeling of the *mlt* intestinal epithelium occurs in the absence of any oncogenic stimuli. Second, invasion in *mlt* is triggered by increased smooth muscle tone rather than qualitative or quantitative changes in matrix proteins. Third, we did not detect tissue stiffening or increased levels of activated Fak in *mlt*, thus arguing that force arising from smooth muscle tension activates a distinct mechanotransductive signaling mechanism than matrix stiffening [62]. Interestingly, metalloproteinases and EMT regulators are upregulated in both the *mlt* intestine [17] and in cancer cells in contact with stiff matrices. Thus, the downstream effects of the different signaling pathways appear to converge in the responding cells.

Although invasive cells in *mlt* are not transformed, they share some features with cancer cells in that they are highly proliferative and not fully differentiated [17]. In contrast, differentiated epithelial cells in homozygous *mlt* larvae rescued by transient Myh11 knockdown have only a modest invasive response to tension [17]. Differentiated epithelial cells in 5 dpf *mlt* heterozygotes also did not respond to menadione, and they formed fewer invadopodia in response to Src than undifferentiated cells. Together, these findings argue that epithelial progenitor cells, and by analogy cancer cells, have an intrinsic capacity for invasive transformation in response to mechanical signals. Supporting this, the invasive response of mature epithelial cells in heterozygous 5 dpf larvae was restored by activation of oncogenic signaling pathways known to play a role in colorectal cancer in humans.

### Extracellular Tension Induces Epithelial Invadopodia-Like Protrusions

In vitro studies link cancer cell invasion to the matrix-degrading function of invadopodia, however it is not known whether invadopodia play a role in cell invasion in vivo [14]. The evidence supporting such a role is principally derived from cell transplantation studies in which tumor cell invasion correlated with Cortactin expression and invadopodia activity [63],[64]. Here we present direct evidence that invadopodia can drive matrix degradation and cell invasion within an intact tissue in vivo. Time lapse movies show that the invadopodia-like protrusions in *mlt* form before the onset of cell invasion, and immunostainings show that the protrusions are located adjacent to degraded segments of the basement membrane, through which the invasive cells migrate into the tissue stroma. The *mlt* protrusions are enriched in the invadopodia proteins Actin, Cortactin, and Src, and their formation requires Tks5, a Src substrate required for invadopodia formation in cancer cells. Trafficking of Mmp14, a metalloproteinase that associates with invadopodia in vitro, was also preferentially distributed to the basal region of *mlt* epithelial cells with protrusions. The latter finding is consistent with our previous work showing upregulated epithelial expression of Mmp14 and Mmp2 in *mlt*, and that metalloproteinase inhibition rescues invasion [17]. All together, these data are, to our knowledge, the most conclusive evidence supporting a role for invadopodia in cell invasion in vivo.

The mechanical properties of cell culture substrates can modify the number and activity of invadopodia that form spontaneously in cancer cells [15],[16]. The findings presented here are novel in that they also show that formation of the invadopodia-like protrusions can be initiated in vivo by activation of a physical signaling mechanism.

### Src Is Sufficient for Formation of Invadopodia-Like Protrusions In Vivo but Not Tissue Cell Invasion

Src signaling is sufficient to induce invadopodia formation in non-transformed mammalian cells [14]. Similarly, activated Src induced formation of invadopodia-like protrusions in wild type zebrafish intestinal epithelial cells. The Src-induced protrusions were located at sites of basement membrane degradation, however cell invasion was never detected in this in vivo model. These findings argue that on their own invadopodia are not sufficient for invasion of mammalian cells from an intact tissue in vivo.

Although activated Src did not induce invasion in wild type epithelial cells, our findings indicate that Src is required for invasion in *mlt*, as the well-characterized Src inhibitor, Src-I1, blocked matrix degradation and invasion in mutant larvae. Interestingly, Src inhibition did not disrupt formation of the invadopodia-like protrusions. The response of *mlt* larvae to Src inhibition therefore resembles the effect of Src knockdown in invasive breast cancer cells [24]. The fact that other Src inhibitors had no effect in *mlt* could be explained by either their inability to access the intestine at larval stages (SU6656) or their different mechanisms of action. Src-l1 competitively inhibits ATP and substrate binding to Src, whereas PP2 only competes for substrate binding [65],[66]. An alternate explanation is that Src-I1 targets other kinases responsible for invasion in *mlt*, however both Src-I1 and PP2 are specific inhibitors of mammalian Src [29], and thus are likely to function comparably to zebrafish Src, which is highly conserved.

Although our data link invasion in *mlt* to Src, we could not detect a higher level of activated Src (phospho-Tyrosine 417) in the *mlt* intestine by Western blot (unpublished data). One explanation for this is that Src and related kinases are already highly activated in larvae due to their function in controlling proliferation and cell polarization [67]. Formation of the protrusions and invasion in *mlt* could therefore involve the relocalization of activated Src to the basal plasma membrane. Alternatively, invasion may require the de novo activation of a relatively small localized pool of cytoplasmic Src. Supporting this idea, force application to the plasma membrane of cultured cells can rapidly activate cytoplasmic Src [68],[69].

### Epithelial Invadopodia and Invasion in *mlt* Are Triggered by a Conserved Redox Signaling Mechanism

We identified a role for redox signaling in *mlt* based on the enhanced expression of ROS sensitive genes, the requirement for Tks5, a component of the ROS producing NAD(P)H oxidase (Nox) complex, and the invasive response of heterozygotes to exogenous oxidative stress induced by ROS generators. Redox signaling has previously been linked to cancer cell invasion and metastasis through formation of invadopodia and activation of EMT regulators [48]–[50],[70]. Our findings are novel in that the *mlt* redox signaling pathway is triggered by a non-cell-autonomous physical signaling mechanism that requires the interaction of cells within adjacent tissue cell layers. A related redox signaling pathway has been implicated in vascular remodeling in mammals [69]. As in *mlt*, contraction of smooth muscle in this model is triggered by mechanical stress applied to the adjacent cell layer (the endothelium) [71],[72]. All together, these findings indicate the presence of an evolutionarily conserved redox-regulated mechanotransductive signaling mechanism that can drive architectural remodeling in diverse vertebrate tissues.

### Myh11, ROS, and Cancer Progression

Recently, heterozygous somatic activating mutations in *MYH11*, similar to *mlt*, were reported in human colorectal cancers [73],[74]. Surprisingly, in the subset of the colorectal cancers examined, the *MYH11* mutations were found more frequently in the epithelium, which does not normally express *MYH11*, than in smooth muscle [68]. The precise role of the *MYH11* mutations in colorectal cancer therefore could not be resolved by these association studies. The findings presented here argue for a primary effect of these mutations in smooth muscle rather than the epithelium. As the invasive remodeling in *mlt* larvae occurs without prior tumor formation, we do not consider *mlt* to be a model for how cancers develop, but rather a model for how cancer cells react to external physical force. Thus, *mlt* can be used to study how epithelial tumors progress from a localized lesion to invasive cancer. Indeed, in one study the presence of the *MYH11* mutations was associated with the invasive transformation of benign adenomatous polyps [74].

The response of heterozygous *mlt* larvae to oxidative stress is an important finding from this study because it provides an example of how tissue architecture can be altered in the appropriate genetic context. Based on our model, we argue that activating mutations of *MYH11* or other human smooth muscle genes could cause invasion of existing cancer in the setting of oxidative stress that is intrinsic to cancers or occurs in the setting of inflammatory conditions that promote cancer formation [75]–[77]. These mutations could alter the function of different types of contractile cells within the tumor stroma besides smooth muscle, such as pericytes and myoepithelial cells, as well stromal fibroblasts that express smooth muscle actin and other contractile proteins. Sustained contraction of these cells could alter extracellular tension in tumors, thus causing them to invade. Alternatively, tonic contraction could also originate from wild type cells present in the tumor stroma such as cancer-associated fibroblasts or related stromal cells.

## Materials and Methods

### Zebrafish

All animals were handled in strict accordance with good animal practice as defined by the relevant national and/or local animal welfare bodies, and all animal work was approved by the animal welfare committee at the University of Pennsylvania School of Medicine. Larvae were raised at 28°C in E3 medium [78] and were staged by age and morphological criteria (size of yolk extension and pigment pattern around yolk extension). Expression of mCherry, mCherry-CAAX, Lifeact-GFP, caSrc, and GFP-KRAS^G12V^ (a generous gift from Steven Leach) in the intestinal epithelium was driven by a 2 kb promoter fragment from the zebrafish *miR194* gene. Expression of GFP in smooth muscle was driven by a promoter fragment from the zebrafish *sm22-alpha* gene [31]. Expression of the ROS sensor Grx1-roGFP2 (a generous gift of Dr. Tobias Dick [54]) in the intestinal epithelium was driven by a promoter fragment from the zebrafish *beta-actin* gene [79]. All fragments were cloned using the multisite gateway system [80],[81]. GFP and mCherry were cloned C-terminal to Src. Zebrafish *axin1* mutants [55] were obtained from the Zebrafish International Resource Center.

### Immunostainings and Confocal Microscopy

3 dpf old larvae were anesthetized with 0.1 mg/ml Tricaine, fixed in 4% PFA/PBS, washed in PBST (PBS+0.1% Tween), dehydrated in methanol, and stored at −20°C. For whole mount staining with anti-laminin and anti-cytokeratin antibodies, larvae were washed in PBST and permeabilized by a 15-min Proteinase K digestion (100 ug/ml in PBST). They were then rinsed in PBST and postfixed in 4% PFA/PBST. The skin above the trunk and intestine was removed using fine forceps. Larvae were stained with antibody in 10% goat serum/PBST. The laminin antibody (Sigma #L-9393) was used at 1∶50 or 1∶200 dilution; the cytokeratin antibody (Thermo Scientific clone AE1/AE3, MS-343-PO) was used at 1∶100 dilution. The Cortactin antibody (Milipore clone 4F11; #05-180) was used at 1∶100. For FAK staining, larvae were dehydrated in −20°C acetone for 30 min followed by staining in 2% BSA/PBST. Anti-phospho-Fak (Y397) (Millipore, #05-1140) was used at 1∶200. Secondary antibodies were labeled with Alexa 568 or 488 (Molecular Probes/Invitrogen). Histological analyses of the mounted specimens were performed as described [17].

Confocal scans of live larvae and sections of immunostained larvae were performed using a Zeiss LSM710 laser scanning microscope. For time lapse microscopy, 3 dpf larvae were anesthetized with 0.1 mg/ml Tricaine [82] and oriented and embedded in 0.8% low melting agarose (Sigma A-9539) in E3/Tricaine heated to 40°C. Analysis were performed at 28°C. Intestines were imaged in two orientations. In tissue cross-sections, the intestine appears as concentric rings of epithelial and surrounding smooth muscle cells. The intestinal lumen and apical surface of the epithelial cells are at the center of the two cellular rings. In sagittal confocal scans of live larvae, the intestine appears as two layers of apposing epithelial cells. A thin layer of smooth muscle cells surrounds the two epithelial cell layers.

To determine the distribution of Mmp14a-mCherry in *mlt* and wild type intestinal epithelial cells, single confocal scans were imported into the ImageJ software. Crops of single cells were copied to a new file and mean mCherry fluorescence was measured in the apical 2/3 and basal 1/3 of each cell. To measure ROS levels in epithelial cells transgenic fish expressing Grx1-roGFP2 were crossed to heterozygous *mlt* fish and their offspring treated with menadione as described below. Larvae mounted for confocal microscopy were excited with 408 and 488 nm lasers, and the emission at 500–530 nm was calculated (as described in [54]). The mean fluorescence emission at each excitation wavelength was calculated for groups of 15–25 cells per larva using the ImageJ software. From this, the 408/488 nm ratio was determined. Statistical analyses were performed using the one-tailed Student's *t* test for data sets with unequal variance.

### Western Blot Analysis

Western blots were performed as previously described [34]. Briefly, equivalent amounts of protein were separated in a 10% SDS–polyacrylamide gel and then electrophoretically transferred to a nitrocellulose membrane using a Bio-Rad protein mini gel apparatus (Bio-Rad Laboratories, Hercules, CA-USA). All protein extracts were prepared from isolated intestines. The membranes were blocked in TBS containing 0.1% Tween 20 and 5% skim milk powder. Blots were probed with anti-Phospho-Caldesmon (Ser789) (Upstate, Lake Placid-NY #07-156), anti-Caldesmon (Calbiochem, Gibbstown, NJ, #ST1109), anti-smMyosin (Biomedical Technologies, Stoughton, Ma #BT-562), anti-smooth muscle Actin (Neomarkers, Fremont, CA, #MS-1296-P0), anti-beta-Actin (Sigma, St. Louis, MO), anti-Phospho-MLC (Ser19) (Cell Signaling, Danvers, MA, #3675), anti-Phospho-ERK1/2 (p44/42) (Thr202/Tyr204) (Cell Signaling, Danvers, MA, #4376), anti-Phospho-p38 (Thr180/Tyr182) (Cell Signaling, Danvers, MA, #4631), anti-Phospho-SAPK/JNK (Thr183/Tyr185) (Cell Signaling, Danvers, MA), and anti-Collagen 1 (NovaTech, France). All blots are representative of at least triplicate experiments.

### Drug Treatments

Larvae were bathed in 1.5 µM Menadione (MP biomedicals) in E3 media for 3 h (3 dpf larvae) or 5 h (5 dpf larvae; 1% DMSO added to E3 media). Larvae were bathed for 3 h in 10 mM l-NAME (Sigma N-5751) dissolved in E3/10 mM Tris pH 7.2.

### RNA In Situ Hybridization

Whole mount in situ hybridization was performed as described [83],[84]. Fragments were cloned into pGem-t-easy (Promega). Double fluorescent in situ hybridization was performed as described [85] using TSA reagents (Perkin-Elmer). Processed specimens were embedded for histological sectioning as previously described [17].

### Morpholino Injection

Morpholinos were injected as described [83]. The *acta2*-morpholino was from Open Biosystems (Morph1609, GCTTTCTTCGTCGTCACACATTTTC, [34]); the sequence of the control morpholino is TGCGCGCCAGACAGGGTGATGAC. Although named “actin, alpha 2, smooth muscle aorta,” *acta2* is expressed in the intestinal smooth muscle [38],[40] and is required for peristaltic contraction [34]. Thus it is the ortholog of mammalian intestinal smooth muscle actin isoform (gamma enteric actin; Actg2) and referred to as *sma*. The h-CaD morpholino (TTATTCCCCTACAAACAGAACTGCA, 1 mM) was designed against the smooth-muscle-specific exon of Caldesmon (based on acc. # BC158175), which was identified from intestinal smooth muscle cDNA. Injection of ∼20 pg caused an in-frame deletion of this exon but had no effect on transcript of the low molecular weight isoform [39]. The morpholino against the translation start site of *tks5* was a generous gift from Danielle Murphy and Sara Courtneidge and was injected as described [25].

### Tissue Stiffness Measurements

The elastic moduli (Young's modulus) of intestines isolated from wild type and *mlt* larvae were measured using a microprobe indenter device [43]. This assay measures the upward force generated by the intestine in response to indentation of the probe applied to the outer (serosal) surface. Tissue compliance (Young's modulus) is the slope of the force versus probe indentation curve. Briefly, a tensiometer probe (Kibron, Inc., Helsinki) with a 100 µm radius flat-bottom needle was mounted on a 3-D micromanipulator with 160 nm step size (Eppendorf, Inc.) attached to an inverted microscope. The tissue was adhered to the bottom of a plastic dish filled with DMEM and imaged by bright field illumination. The bottom of the probe was brought through the air-water interface until it rested at the surface of the cylindrical tissue with a diameter of approximately 80 microns. The probe was calibrated using the known surface tension of a pure water/air interface, and the stress applied by the tissue to the probe as it was lowered was measured as a function of indentation depth. In principle, this deformation geometry is that of an infinite plane compressing a cylindrical object, and the absolute values of elastic modulus can be calculated from appropriate models that require assumptions about the adherence of the tissue to the probe and the glass slide, whether the sample is modeled as a uniform cylinder or an elastic shell, and other structural factors that confound calculation of the absolute value of elastic modulus from the force-indentation data. In this study the primary interest is in the relative stiffness of wild type and mutant tissue, and therefore we present only the primary data, which consists of the elastic resistance of the tissues as a function of indentation depth. Indentations (≥13 per intestine) spanned the range from 160 nm, which would measure small strain reflecting linear elasticity to indentations, up to 20 microns, which would reveal differences in large strain deformation or rupture. After the largest indentations, measurements were repeated at small strains to confirm that the deformations were recoverable. For statistical analyses, a one-tailed Student's *t* test for data sets with unequal variance was performed to determine the significance of differences between Young's moduli of wild type and *mlt* intestines samples.

### Transcriptional Profiling

RNA was isolated from trunk sections of 74 hpf larvae that encompassed the mid- and posterior intestine. Six pairs of mutants and wild type siblings were analyzed (25–35 trunks per sample). RNA was recovered using Trizol (Invitrogen) and Qiagen RNeasy columns. Probes were hybridized to the Affymetrix zebrafish genome array (Affymetrix #900487). The array data were evaluated by importing affymetrix *.cel files into genespring v.7.3.1, and expression intensities were calculated using gcrma for each probe set. Gene expression data were validated by quantitative RT-PCR using sybr green and by RNA in-situ hybridization.

### Quantitative Real-Time PCR

RNA was recovered from intestines manually dissected from larvae using Trizol and Qiagen RNeasy columns. For each PCR amplification RNA from 15 or more larvae was pooled. An amplified fragment from the *tata-box binding protein* cDNA (*tbp*) was used as internal standard. PCR amplification and data analysis were performed as described [86] using sybr green (Applied Biosystems, UK). Statistical analyses were performed using the one-tailed Student's *t* test for data sets with unequal variance.

### PCR Primers

*glutathione peroxidase*: 5′ gctgttcagcctggactttt; 3′ QPCR cgttgctgagtttggactttt; 3′ in situ ctcagatgaacgagctgcac

*jun B*: 5′ tctgttgggttacggtcaca; 3′ QPCR cgtctggatgatgagcctct; 3′ in-situ ggaccttctgcttgagttgc

src (including attB sites): 5′ ggggacaagtttgtacaaaaaagcaggctgccaccatgggtggagtcaagagtaa; 3′ ggggaccactttgtacaagaaagctgggtcgaggttttctccgggttggta; 3′ Y528F ggggaccactttgtacaagaaagctgggtcgaggttttctccgggttggaattgtggttc.

### Cell Dissociation Experiments

6 hpf (shield stage) zebrafish embryos were sterilized by two 10-min washes with 0.1% sodium hypochlorite and raised in sterile embryo medium. Intestines were dissected from sterilized embryos at 74 hpf and dissociated in 0.25% trypsin/EDTA. Cells were collected by centrifugation for 5 min at 800 g and treated with 1.5 µM menadione for 1 h in DMEM supplemented with 10% FBS, 100 ug/ul pen/strep, and 0.2% amphotericin B. Treated cells were centrifuged and immediately fixed in sample buffer and stored at −80°C for Western blots.

## Supporting Information

Figure S1 *tks5* knockdown rescues epithelial invasion in the intestine of *mlt* mutant larvae. (A–C) Lateral images of live 5 dpf larvae. Control morpholino injected *mlt* larvae show cystic expansion of the intestinal epithelium (intestinal epithelium outlined in red; lateral view) (B), while only a small number of cysts can still be detected in the *tks5* morpholino injected *mlt* larvae (D, arrowhead). The majority of the posterior intestine in these larvae resembles WT (A, C). Findings confirmed histologically (not shown; n = 6 *mlt* larvae).
(PDF)

Figure S2 Src induces less invadopodia-like protrusions in mature intestinal epithelial cells than in epithelial progenitor cells. (A) Sagittal confocal scans through the intestine of a 5 dpf *Tg(miR194:Lifeact-GFP)* with mosaic expression of a caSrc-mCherry transgene. Only a small number of invadopodia-like protrusions are detected (green; arrowheads). (B) Quantification showed that invadopodia-like protrusions were detected in 89% of caSRC-mCherry positive cells in 3 dpf larvae (*n* = 28 cells in 5 larvae), while they were present in only 9% of cells in 5 dpf larvae (*n* = 64 cells in 5 larvae). Error bars, standard deviation.
(PDF)

Figure S3 Smooth muscle actin (*sma*) knockdown rescues *mlt* mutants. (A–C) Western blot showing reduced Sma protein in the intestine of 74 hpf and 4 dpf Sma MO injected larvae (lane 2, 4) compared to a control MO (lane 1, 3). Myh11 protein levels are unaffected by the Sma knockdown (lanes 1–4). Each lane contains protein extracted from the intestines dissected from 30 larvae. This blot is representative of two independent experiments.
(PDF)

Figure S4 h-CaD knockdown triggers invasion in *mlt* heterozygotes. Lateral views of 5 dpf WT (A) and *mlt/+* (B) larvae that had been injected with a splice-blocking morpholino (MO) that specifically targets h-CaD. Intestinal morphology is normal in the WT larva, whereas there is cystic expansion of the *mlt/+* intestine. Histological analyses confirmed invasive expansion of the *mlt/+* intestine (Figure 5D and 5E). Invasion was detected in 69% larvae from a *mlt/+* intercross (*n* = 240) of which 66% were predicted to be *mlt/+*. 12 of 12 genotyped larvae were *mlt/+*.
(PDF)

Figure S5 Invasive remodeling occurs without changes in matrix composition or FAK activation. (A) Western blot showing normal levels of Type 1 Collagen (Col1) and Fibronectin (Fn) in the intestine of 74 hpf *mlt* larvae compared with WT. beta-Actin serves as loading control. (B–D) Histological sections show no activation of FAK by phosphorylation (p-FAK, green) in the epithelium of WT or *mlt* larvae at 74 hpf. Basement membrane detected with anti-laminin staining (red) (asterisk, stratification; arrowheads, invasive sites). (E) Lower power image showing expected p-FAK (green) in the myoseptum of a WT larva.
(PDF)

Figure S6 AP-1 transcription factors, ROS responsive genes, and MAP-Kinase signaling are activated in the *mlt* intestine. (A–D) Whole mount in situ hybridization shows strong expression (blue) of the AP-1 gene *junB* and the ROS activated *gene gluthatione peroxidase* (*gpx*) in the intestine of 74 hpf *mlt* (B, D) but not WT (A, C) larvae. *n* = 15 *mlt* and wild type larvae examined. (E, F) Histological cross-sections of whole mount specimens processed for fluorescent RNA in situ hybridization show strong *junB* expression (green) in *mlt* intestinal epithelial cells with only low level expression in smooth muscle cells (labeled red by *myh11* expression). (G, H) Similarly, *gpx* expression (green) can only be detected in the *mlt* intestinal epithelium. *n* = 12 *mlt* and 12 wild type larvae examined. (I) Quantitative RT-PCR shows increased *junB* expression in the intestine of *mlt* homozygotes. *junB* expression is also increased in menadione treated wild type larvae (+/+), and to a greater degree in *mlt* heterozygous larvae (versus untreated wild type; contr). (J) Western blot showing phosphorylation of several components of the Map-Kinase signaling pathway in intestines dissected from *mlt* larvae at 74 hpf and 96 hpf. ERK is prematurely activated at 74 hpf in *mlt*. p38 (Mapk), Jnk, and Sapk are strongly activated in *mlt* but not WT larvae at 74 hpf and 96 hpf. beta-Actin, loading control. Western blots are representative of between two and four independent experiments.
(PDF)

Figure S7 Comparable induction of *gpx* expression in WT larvae that express caSrc and homozygous *mlt* larvae. (A–C) Whole mount RNA in situ hybridization for *gluthatione peroxidase* expression (*gpx*, blue). *gpx* expression is low in the WT intestine (A), whereas it is markedly elevated in the *mlt* intestine (B; see also Figures 6C, 6D, S9G, and S9H). *n* = 15 *mlt* and 15 wild type larvae. (C) Mosaic expression of a caSrc transgene induces *gpx* expression in the wild type intestine (C, brackets and arrows). Interestingly, expression of caSrc in the pronephric duct (via the *miR194* promoter) also induces *gpx* expression (C, arrowhead). *n* = 12 larvae.
(PDF)

Movie S1 Epithelial actin cytoskeleton in the wild type larval intestine. Sagittal 3-D renderings through the posterior intestine of a 5 dpf wild type Lifeact-GFP transgenic larva. Lifeact-GFP is principally located in the brush border of the epithelial cell apical plasma membrane.
(AVI)

Movie S2 Invadopodia-like protrusions arise from the basal intestinal epithelial cell plasma membrane of mlt larvae. Sagittal 3-D renderings through the posterior intestine of a 5 dpf mlt Lifeact-GFP transgenic larva. In mlt epithelial cells Lifeact-GPF labels basal invadopodia-like protrusions in addition to the apical plasma membrane.
(AVI)

Movie S3 Formation of actin-rich invadopodia-like basal membrane protrusions in *mlt* epithelial cells. Sagittal confocal scans through the intestine of *mlt* larva expressing Lifeact-GFP in the intestinal epithelium (*Tg(miR194:Lifeact-GFP)*), beginning at 72 hpf. Initially, Lifeact-GFP (green) bound to Actin is only present in the epithelial cell apical brush border. As development proceeds, Actin-rich invadopodia-like protrusions form at the basal epithelial cell membrane and persist for several hours. Time lapse movie; 1 s corresponds to 15 min real time; duration is 5 h.
(AVI)

Movie S4 Formation of actin-rich invadopodia-like protrusions precedes invasion of mlt epithelial cells. Sagittal confocal scans through the intestine of mlt larva expressing Lifeact-GFP in the intestinal epithelium (Tg(miR194:Lifeact-GFP)), beginning at 76 hpf. Actin rich protrusions are present at the basal membrane, and the protrusions frequently precede the development of invasive lesions (arrows). Time lapse movie; 1 s corresponds to 15 min real time; duration is 5 h.
(AVI)

Movie S5 Formation of actin-rich invadopodia-like protrusions precedes invasion of mlt epithelial cells. Sagittal confocal scans through the intestine of mlt larva expressing Lifeact-GFP in the intestinal epithelium (Tg(miR194:Lifeact-GFP)), beginning at 74 hpf. Actin-rich protrusions are present at the basal membrane, and the protrusions frequently precede the development of invasive lesions (arrows). Time lapse movie; 1 s corresponds to 15 min real time; duration is 5 h.
(AVI)

Movie S6 Lifeact-GFP localization in the WT intestinal epithelium. Sagittal confocal scans through the intestine of WT larva expressing Lifeact-GFP in the intestinal epithelium (*Tg(miR194:Lifeact-GFP)*), beginning at 74 hpf. Actin bound Lifeact-GFP is localized to the epithelial cell apical brush border membrane (adjacent to the intestinal lumen). 1 s corresponds to 15 min real time, duration is 5 h.
(AVI)

Movie S7 Peristaltic circular smooth muscle contractions in the 76 hpf WT intestine. Sagittal confocal scans through the intestine of a 76 hpf WT larva expressing GFP (green) in smooth muscle cells and mCherry (red) in the intestinal epithelium (*Tg(miR194:mCherry; Sm22a:GFP)*). Time lapse movie; 1 s corresponds to 9 min real time.
(MOV)

Movie S8 Constitutive smooth muscle contraction in the 76 hpf *mlt* intestine. Sagittal confocal scans through the intestine of a 76 hpf *mlt* larva expressing GFP (green) in smooth muscle cells and mCherry (red) in the intestinal epithelium (*Tg(miR194:mCherry; Sm22a:GFP)*). Slow tonic contraction of the intestinal smooth muscle disrupts intestinal architecture. Time lapse movie; 1 s corresponds to 9 min real time.
(MOV)

Movie S9 Absence of smooth muscle contraction in the 72 hpf WT intestine. Sagittal confocal scans through the intestine of a 72 hpf WT larva expressing GFP (green) in smooth muscle cells and mCherry (red) in the intestinal epithelium (*Tg(miR194:mCherry; Sm22a:GFP)*). Time lapse movie; 1 s corresponds to 9 min real time.
(MOV)

Movie S10 Absence of smooth muscle contraction in the 72 hpf *mlt* intestine. Sagittal confocal scans through the intestine of a 72 hpf *mlt* larva expressing GFP (green) in smooth muscle cells and mCherry (red) in the intestinal epithelium (*Tg(miR194:mCherry; Sm22a:GFP)*). Epithelial cell invasion is already present at this stage. Time lapse movie; 1 s corresponds to 9 min real time.
(MOV)

Table S1 Transcriptional profiling identifies genes with increased expression in *mlt* (1.5-fold or greater). Array signal ratio (*mlt*:WT) shown in Column C; data are mean of six experiments. Gene expression ratio (*mlt*:WT) as measured by quantitative PCR, Column D (74 hpf) and E (80 hpf). EST annotation of microarray probe (if different from Genbank accession number, Column B).
(XLS)

## Abbreviations

caSrc: Src-Y528F
dpf: days post fertilization
h-Cad: smooth muscle isoform of Caldesmon
hpf: hours post fertilization
*mlt*: *meltdown*
ROS: reactive oxygen species
*sma*: *smooth muscle actin*
WT: wildtype

## References

[1] de Rooij J, Kerstens A, Danuser G, Schwartz MA, Waterman-Storer CM (2005) Integrin-dependent actomyosin contraction regulates epithelial cell scattering. J Cell Biol 171: 153–164. 16216928 10.1083/jcb.200506152 PMC2171213

[2] Yeung T, Georges PC, Flanagan LA, Marg B, Ortiz M, et al (2005) Effects of substrate stiffness on cell morphology, cytoskeletal structure, and adhesion. Cell Motil Cytoskeleton 60: 24–34. 15573414 10.1002/cm.20041

[3] Paszek MJ, Zahir N, Johnson KR, Lakins JN, Rozenberg GI, et al (2005) Tensional homeostasis and the malignant phenotype. Cancer Cell 8: 241–254. 16169468 10.1016/j.ccr.2005.08.010

[4] Papusheva E, Heisenberg CP (2010) Spatial organization of adhesion: force-dependent regulation and function in tissue morphogenesis. EMBO J 29 (16) 2753–2768. 20717145 10.1038/emboj.2010.182 PMC2924654

[5] Lehoux S, Tedgui A (2003) Cellular mechanics and gene expression in blood vessels. J Biomech 36: 631–643. 12694993 10.1016/s0021-9290(02)00441-4

[6] Birukov KG (2009) Cyclic stretch, reactive oxygen species, and vascular remodeling. Antioxid Redox Signal 11: 1651–1667. 19186986 10.1089/ars.2008.2390 PMC2842585

[7] Levental KR, Yu H, Kass L, Lakins JN, Egeblad M, et al (2009) Matrix crosslinking forces tumor progression by enhancing integrin signaling. Cell 139: 891–906. 19931152 10.1016/j.cell.2009.10.027 PMC2788004

[8] Samuel MS, Lopez JI, McGhee EJ, Croft DR, Strachan D, et al (2011) Actomyosin-mediated cellular tension drives increased tissue stiffness and β-catenin activation to induce epidermal hyperplasia and tumor growth. Cancer Cell 19: 776–791. 21665151 10.1016/j.ccr.2011.05.008 PMC3115541

[9] Whitehead J, Vignjevic D, Futterer C, Beaurepaire E, Robine S, Farge E (2008) Mechanical factors activate beta-catenin-dependent oncogene expression in APC mouse colon. HFSP J 2: 286–294. 19404440 10.2976/1.2955566 PMC2639941

[10] Boucher Y, Jain RK (1992) Microvascular pressure is the principal driving force for interstitial hypertension in solid tumors: implications for vascular collapse. Cancer Res 52: 5110–5114. 1516068

[11] Padera TP, Stoll BR, Tooredman JB, Capen D, di Tomaso E, Jain RK (2004) Pathology: cancer cells compress intratumour vessels. Nature 427 (6976) 695. 14973470 10.1038/427695a

[12] Craig DH, Basson MD (2009) Biological impact of mechanical stimuli on tumor metastasis. Cell Cycle 8 (6) 828–831. 19229129 10.4161/cc.8.6.7940 PMC5700767

[13] Weaver AM (2006) Invadopodia: specialized cell structures for cancer invasion. Clin Exp Metastasis 23: 97–105. 16830222 10.1007/s10585-006-9014-1

[14] Murphy DA, Courtneidge SA (2011) The “ins” and “outs” of podosomes and invadopodia: characteristics, formation and function. Nat Rev Mol Cell Biol 12: 413–426. 21697900 10.1038/nrm3141 PMC3423958

[15] Alexander NR, Branch KM, Parekh A, Clark ES, Iwueke IC, et al (2008) Extracellular matrix rigidity promotes invadopodia activity. Curr Biol 18: 1295–1299. 18718759 10.1016/j.cub.2008.07.090 PMC2555969

[16] Parekh A, Ruppender NS, Branch KM, Sewell-Loftin MK, Lin J, et al (2011) Sensing and modulation of invadopodia across a wide range of rigidities. Biophys J 100: 573–582. 21281571 10.1016/j.bpj.2010.12.3733 PMC3030182

[17] Wallace KN, Dolan AC, Seiler C, Smith EM, Yusuff S, et al (2005) Mutation of smooth muscle myosin causes epithelial invasion and cystic expansion of the zebrafish intestine. Dev Cell 8: 717–726. 15866162 10.1016/j.devcel.2005.02.015

[18] Seals DF, Azucena EF Jr, Pass I, Tesfay L, Gordon R, Woodrow M, Resau JH, Courtneidge SA (2005) The adaptor protein Tks5/Fish is required for podosome formation and function, and for the protease-driven invasion of cancer cells. Cancer Cell 7: 155–165. 15710328 10.1016/j.ccr.2005.01.006

[19] Courtneidge SA, Azucena EF, Pass I, Seals DF, Tesfay L (2005) The SRC substrate Tks5, podosomes (invadopodia), and cancer cell invasion. Cold Spring Harb Symp Quant Biol 70: 167–171. 16869750 10.1101/sqb.2005.70.014

[20] Crimaldi L, Courtneidge SA, Gimona M (2009) Tks5 recruits AFAP-110, p190RhoGAP, and cortactin for podosome formation. Exp Cell Res 315 (15) 2581–2592. 19540230 10.1016/j.yexcr.2009.06.012

[21] Wallace KN, Akhter S, Smith EM, Lorent K, Pack M (2005) Intestinal growth and differentiation in zebrafish. Mech Dev 122 (2) 157–173. 15652704 10.1016/j.mod.2004.10.009

[22] Caldieri G, Ayala I, Attanasio F, Buccione R (2009) Cell and molecular biology of invadopodia. Int Rev Cell Mol Biol 275: 1–34. 19491051 10.1016/S1937-6448(09)75001-4

[23] Riedl J, Crevenna AH, Kessenbrock K, Yu JH, Neukirchen D, et al (2008) Lifeact: a versatile marker to visualize F-actin. Nat Methods 5: 605–607. 18536722 10.1038/nmeth.1220 PMC2814344

[24] Mader CC, Oser M, Magalhaes MA, Bravo-Cordero JJ, Condeelis J, Koleske AJ, Gil-Henn H (2011) An EGFR-Src-Arg-cortactin pathway mediates functional maturation of invadopodia and breast cancer cell invasion. Cancer Res 71 (5) 1730–1741. 21257711 10.1158/0008-5472.CAN-10-1432 PMC3057139

[25] Murphy DA, Bromann BA, Tsai PAA, Kawakami JHA, Maurer YA, et al (2011a) A Src-Tks5 pathway is required for neural crest cell migration during embryonic development. PLoS ONE 6: e22499 doi:10.1371/journal. pone.0022499. 21799874 10.1371/journal.pone.0022499 PMC3143166

[26] Chen WT, Chen JM, Parsons SJ, Parsons JT (1985) Local degradation of fibronectin at sites of expression of the transforming gene product pp60src. Nature 316: 156–158. 2989711 10.1038/316156a0

[27] Chen W T (1989) Proteolytic activity of specialized surface protrusions formed at rosette contact sites of transformed cells. Journal of Experimental Zoology 251: 167–185. 2549171 10.1002/jez.1402510206

[28] Kelley LC, Ammer AG, Hayes KE, Martin KH, Machida K, et al (2010) Oncogenic Src requires a wild-type counterpart to regulate invadopodia maturation. J Cell Sci 123: 3923–3932. 20980387 10.1242/jcs.075200 PMC2972274

[29] Bain J, Plater L, Elliott M, Shpiro N, Hastie CJ, McLauchlan H, Klevernic I, Arthur JS, Alessi DR, Cohen P (2007) The selectivity of protein kinase inhibitors: a further update. Biochem J 408 (3) 297–315. 17850214 10.1042/BJ20070797 PMC2267365

[30] Molina GA, Watkins SC, Tsang M (2007) Generation of FGF reporter transgenic zebrafish and their utility in chemical screens. BMC Dev Biol 7: 62. 17553162 10.1186/1471-213X-7-62 PMC1904198

[31] Seiler C, Abrams J, Pack M (2010) Characterization of zebrafish intestinal smooth muscle development using a novel sm22α-b promoter. Dev Dyn 239: 2806–2812. 20882680 10.1002/dvdy.22420 PMC4739357

[32] North AJ, Gimona M, Lando Z, Small JV (1994) Actin isoform compartments in chicken gizzard smooth muscle cells. J Cell Sci 107 (Pt 3) 445–455. 8006065 10.1242/jcs.107.3.445

[33] Holmberg A, Olsson C, Hennig GW (2007) TTX-sensitive and TTX-insensitive control of spontaneous gut motility in the developing zebrafish (Danio rerio) larvae. J Exp Biol 210 (Pt 6) 1084–1091. 17337720 10.1242/jeb.000935

[34] Davuluri G, Seiler C, Abrams J, Soriano AJ, Pack M (2010) Differential effects of thin and thick filament disruption on zebrafish smooth muscle regulatory proteins. Neurogastroenterol Motil 22: 1100–e285. 20591105 10.1111/j.1365-2982.2010.01545.x PMC3902778

[35] Ding HL, Ryder JW, Stull JT, Kamm KE (2009) Signaling processes for initiating smooth muscle contraction upon neural stimulation. J Biol Chem 284: 15541–15548. 19349274 10.1074/jbc.M900888200 PMC2708850

[36] Kordowska J, Huang R, Wang CL (2006) Phosphorylation of caldesmon during smooth muscle contraction and cell migration or proliferation. J Biomed Sci 13: 159–172. 16453176 10.1007/s11373-005-9060-8

[37] Akata T (2007) Cellular and molecular mechanisms regulating vascular tone. Part 2: regulatory mechanisms modulating Ca2+ mobilization and/or myofilament Ca2+ sensitivity in vascular smooth muscle cells. J Anesth 21: 232–242. 17458653 10.1007/s00540-006-0488-4

[38] Smolock EM, Trappanese DM, Chang S, Wang T, Titchenell P, Moreland RS (2009) siRNA-mediated knockdown of h-caldesmon in vascular smooth muscle. Am J Physiol Heart Circ Physiol 297: H1930–H1939. 19767533 10.1152/ajpheart.00129.2009 PMC2781382

[39] Abrams J, Davuluri G, Seiler C, Pack M (2012) Smooth muscle caldesmon modulates peristalsis in the wild type and non-innervated zebrafish. Intestine Neurogastroenterology and Motility 24 (3) 288–299. 22316291 10.1111/j.1365-2982.2011.01844.x PMC3919438

[40] Georgijevic S, Subramanian Y, Rollins EL, Starovic-Subota O, Tang AC, Childs SJ (2007) Spatiotemporal expression of smooth muscle markers in developing zebrafish gut. Dev Dyn 236: 1623–1632. 17474123 10.1002/dvdy.21165

[41] Earley JJ, Su X, Moreland RS (1998) Caldesmon inhibits active crossbridges in unstimulated vascular smooth muscle: an antisense oligodeoxynucleotide approach. Circ Res 83: 661–667. 9742062 10.1161/01.res.83.6.661

[42] Holmberg A, Olsson C, Holmgren S (2006) The effects of endogenous and exogenous nitric oxide on gut motility in zebrafish Danio rerio embryos and larvae. J Exp Biol 209: 2472–2479. 16788030 10.1242/jeb.02272

[43] Levental I, Levental KR, Klein EA, Assoian R, Miller RT, et al (2010) A simple indentation device for measuring micrometer-scale tissue stiffness. Journal of Physics: Condensed Matter 22: 194120. 21386443 10.1088/0953-8984/22/19/194120 PMC3392911

[44] Snow CJ, Henry CA (2009) Dynamic formation of microenvironments at the myotendinous junction correlates with muscle fiber morphogenesis in zebrafish. Gene Expr Patterns 9: 37–42. 18783736 10.1016/j.gep.2008.08.003 PMC2655214

[45] Bergman MR, Cheng S, Honbo N, Piacentini L, Karliner JS, Lovett DH (2003) A functional activating protein 1 (AP-1) site regulates matrix metalloproteinase 2 (MMP-2) transcription by cardiac cells through interactions with JunB-Fra1 and JunB-FosB heterodimers. Biochem J 369: 485–496. 12371906 10.1042/BJ20020707 PMC1223099

[46] Ozanne BW, Spence HJ, McGarry LC, Hennigan RF (2007) Transcription factors control invasion: AP-1 the first among equals. Oncogene 26: 1–10. 16799638 10.1038/sj.onc.1209759

[47] Radisky DC, Levy DD, Littlepage LE, Liu H, Nelson CM, et al (2005) Rac1b and reactive oxygen species mediate MMP-3-induced EMT and genomic instability. Nature 436: 123–127. 16001073 10.1038/nature03688 PMC2784913

[48] Cannito S, Novo E, di Bonzo LV, Busletta C, Colombatto S, Parola M (2010) Epithelial–mesenchymal transition: from molecular mechanisms, redox regulation to implications in human health and disease. Antioxid Redox Signal 12: 1383–1430. 19903090 10.1089/ars.2009.2737

[49] Diaz B, Shani G, Pass I, Anderson D, Quintavalle M, Courtneidge SA (2009) Tks5-dependent, nox-mediated generation of reactive oxygen species is necessary for invadopodia formation. Sci Signal 2: ra53. 19755709 10.1126/scisignal.2000368 PMC2810640

[50] Gianni D, Diaz B, Taulet N, Fowler B, Courtneidge SA, Bokoch GM (2009) Novel p47(phox)-related organizers regulate localized NADPH oxidase 1 (Nox1) activity. Sci Signal 2: ra54. 19755710 10.1126/scisignal.2000370 PMC2850287

[51] Weaver AM (2009) Regulation of cancer invasion by reactive oxygen species and Tks family scaffold proteins. Sci Signal 2: pe56. 19755707 10.1126/scisignal.288pe56 PMC2893140

[52] Hasegawa T, Bando A, Tsuchiya K, Abe S, Okamoto M, et al (2004) Enzymatic and nonenzymatic formation of reactive oxygen species from 6-anilino-5,8-quinolinequinone. Biochim Biophys Acta 1670: 19–27. 14729138 10.1016/j.bbagen.2003.10.008

[53] Chuang YY, Chen Y, Gadisetti Chandramouli VR, Cook JA, et al (2002) Gene expression after treatment with hydrogen peroxide, menadione, or t-butyl hydroperoxide in breast cancer cells. Cancer Res 62: 6246–6254. 12414654

[54] Gutscher M, Pauleau AL, Marty L, Brach T, Wabnitz GH, Samstag Y, Meyer AJ, Dick TP (2008) Real-time imaging of the intracellular glutathione redox potential. Nat Methods 5 (6) 553–559. 18469822 10.1038/nmeth.1212

[55] Heisenberg CP, Houart C, Take-Uchi M, Rauch GJ, Young N, et al (2001) A mutation in the Gsk3-binding domain of zebrafish Masterblind/Axin1 leads to a fate transformation of telencephalon and eyes to diencephalon. Genes Dev 15: 1427–1434. 11390362 10.1101/gad.194301 PMC312705

[56] Le X, Langenau DM, Keefe MD, Kutok JL, Neuberg DS, Zon LI (2007) Heat shock-inducible Cre/Lox approaches to induce diverse types of tumors and hyperplasia in transgenic zebrafish. Proc Natl Acad Sci U S A 104: 9410–9415. 17517602 10.1073/pnas.0611302104 PMC1890508

[57] Cheesman SE, Neal JT, Mittge E, Seredick BM, Guillemin K (2011) Epithelial cell proliferation in the developing zebrafish intestine is regulated by the Wnt pathway and microbial signaling via Myd88. Proc Natl Acad Sci U S A 108: 4570–4577. 20921418 10.1073/pnas.1000072107 PMC3063593

[58] Phelps RA, Chidester S, Dehghanizadeh S, Phelps J, Sandoval IT, et al (2009) A two-step model for colon adenoma initiation and progression caused by APC loss. Cell 137: 623–634. 19450512 10.1016/j.cell.2009.02.037 PMC2706149

[59] Mammoto T, Ingber DE (2010) Mechanical control of tissue and organ development. Development 137: 1407–1420. 20388652 10.1242/dev.024166 PMC2853843

[60] Janmey PA, Miller RT (2011) Mechanisms of mechanical signaling in development and disease. J Cell Sci 2011 124 (Pt 1) 9–18. 10.1242/jcs.071001 PMC3001405 21172819

[61] Wirtz D, Konstantopoulos K, Searson PC (2011) The physics of cancer: the role of physical interactions and mechanical forces in metastasis. Nat Rev Cancer 11 (7) 512–522. 21701513 10.1038/nrc3080 PMC3262453

[62] Tee SY, Bausch AR, Janmey PA (2009) The mechanical cell. Curr Biol 19: R745–R748. 19906576 10.1016/j.cub.2009.06.034 PMC2888099

[63] Clark ES, Brown B, Whigham AS, Kochaishvili A, Yarbrough WG, Weaver AM (2009) Aggressiveness of HNSCC tumors depends on expression levels of cortactin, a gene in the 11q13 amplicon. Oncogene 28: 431–444. 18931703 10.1038/onc.2008.389 PMC2709457

[64] Blouw B, Seals DF, Pass I, Diaz B, Courtneidge SA (2008) A role for the podosome/invadopodia scaffold protein Tks5 in tumor growth in vivo. Eur J Cell Biol 87: 555–567. 18417249 10.1016/j.ejcb.2008.02.008 PMC2629379

[65] Karni R, Mizrachi S, Reiss-Sklan E, Gazit A, Livnah O, Levitzki A (2003) The pp60c-Src inhibitor PP1 is non-competitive against ATP. FEBS Lett 537 (1–3) 47–52. 12606029 10.1016/s0014-5793(03)00069-3

[66] Tian G, Cory M, Smith AA, Knight WB (2001) Structural determinants for potent, selective dual site inhibition of human pp60c-src by 4-anilinoquinazolines. Biochemistry 40 (24) 7084–7091. 11401553 10.1021/bi0100586

[67] Guarino M (2010) Src signaling in cancer invasion. J Cell Physiol 223: 14–26. 20049846 10.1002/jcp.22011

[68] Wang Y, Botvinick EL, Zhao Y, Berns MW, Usami S, Tsien RY, Chien S (2005) Visualizing the mechanical activation of Src. Nature 434 (7036) 1040–1045. 15846350 10.1038/nature03469

[69] Na S, Collin O, Chowdhury F, Tay B, Ouyang M, et al (2008) Rapid signal transduction in living cells is a unique feature of mechanotransduction. Proc Natl Acad Sci U S A 105: 6626–6631. 18456839 10.1073/pnas.0711704105 PMC2373315

[70] Behrend L, Henderson G, Zwacka RM (2003) Reactive oxygen species in oncogenic transformation. Biochem Soc Trans 31: 1441–1444. 14641084 10.1042/bst0311441

[71] Ando J, Yamamoto K (2011) Effects of shear stress and stretch on endothelial function. Antioxid Redox Signal 15 (5) 1389–1403. 20854012 10.1089/ars.2010.3361

[72] Paravicini TM, Touyz RM (2006) Redox signaling in hypertension. Cardiovasc Res 71: 247–258. 16765337 10.1016/j.cardiores.2006.05.001

[73] Alhopuro P, Phichith D, Tuupanen S, Sammalkorpi H, Nybondas M, et al (2008) Unregulated smooth-muscle myosin in human intestinal neoplasia. Proc Natl Acad Sci U S A 105: 5513–5518. 18391202 10.1073/pnas.0801213105 PMC2291082

[74] Vickaryous N, Polanco-Echeverry G, Morrow S, Suraweera N, Thomas H, et al (2008) Smooth-muscle myosin mutations in hereditary non-polyposis colorectal cancer syndrome. Br J Cancer 99: 1726–1728. 18941465 10.1038/sj.bjc.6604737 PMC2584943

[75] Clevers H (2004) At the crossroads of inflammation and cancer. Cell 118: 671–674. 15369667 10.1016/j.cell.2004.09.005

[76] Kim BG, Li C, Qiao W, Mamura M, Kasprzak B, et al (2006) Smad4 signalling in T cells is required for suppression of gastrointestinal cancer. Nature 441: 1015–1019. 16791201 10.1038/nature04846

[77] Fox JG, Wang TC (2007) Inflammation, atrophy, and gastric cancer. J Clin Invest 117: 60–69. 17200707 10.1172/JCI30111 PMC1716216

[78] Westerfield M (M) (1995) The zebrafish book: a guide for the laboratory use of zebrafish (danio rerio). University of Oregon Press.

[79] Higashijima S, Okamoto H, Ueno N, Hotta Y, Eguchi G (1997) High-frequency generation of transgenic zebrafish which reliably express GFP in whole muscles or the whole body by using promoters of zebrafish origin. Dev Biol 192 (2) 289–299. 9441668 10.1006/dbio.1997.8779

[80] Villefranc JA, Amigo J, Lawson ND (2007) Gateway compatible vectors for analysis of gene function in the zebrafish. Dev Dyn 236: 3077–3087. 17948311 10.1002/dvdy.21354 PMC4518551

[81] Kwan KM, Fujimoto E, Grabher C, Mangum BD, Hardy ME, et al (2007) The Tol2kit: a multisite gateway-based construction kit for Tol2 transposon transgenesis constructs. Dev Dyn 236: 3088–3099. 17937395 10.1002/dvdy.21343

[82] Rombough PJ (2007) Ontogenetic changes in the toxicity and efficacy of the anaesthetic MS222 (tricaine methanesulfonate) in zebrafish (Danio rerio) larvae. Comp Biochem Physiol A Mol Integr Physiol 148: 463–469. 17643329 10.1016/j.cbpa.2007.06.415

[83] Seiler C, Finger-Baier KC, Rinner O, Makhankov YV, Schwarz H, et al (2005) Duplicated genes with split functions: independent roles of protocadherin15 orthologues in zebrafish hearing and vision. Development 132: 615–623. 15634702 10.1242/dev.01591

[84] Kishimoto Y, Lee KH, Zon L, Hammerschmidt M, Schulte-Merker S (1997) The molecular nature of zebrafish swirl: BMP2 function is essential during early dorsoventral patterning. Development 124: 4457–4466. 9409664 10.1242/dev.124.22.4457

[85] Jülich D, Hwee Lim C, Round J, Nicolaije C, Schroeder J, et al (2005) beamter/deltaC and the role of Notch ligands in the zebrafish somite segmentation, hindbrain neurogenesis and hypochord differentiation. Dev Biol 286: 391–404. 16125692 10.1016/j.ydbio.2005.06.040

[86] Matthews RP, Lorent K, Russo P, Pack M (2004) The zebrafish onecut gene hnf-6 functions in an evolutionarily conserved genetic pathway that regulates vertebrate biliary development. Dev Biol 274: 245–259. 15385156 10.1016/j.ydbio.2004.06.016

