# Supplementary figures and images for: Smooth Muscle Tension Induces Invasive Remodeling of the Zebrafish Intestine
Source: PLoS Biol. 2012 Sep 4;10(9):e1001386. doi: 10.1371/journal.pbio.1001386 (PMC3433428; doi:10.1371/journal.pbio.1001386)

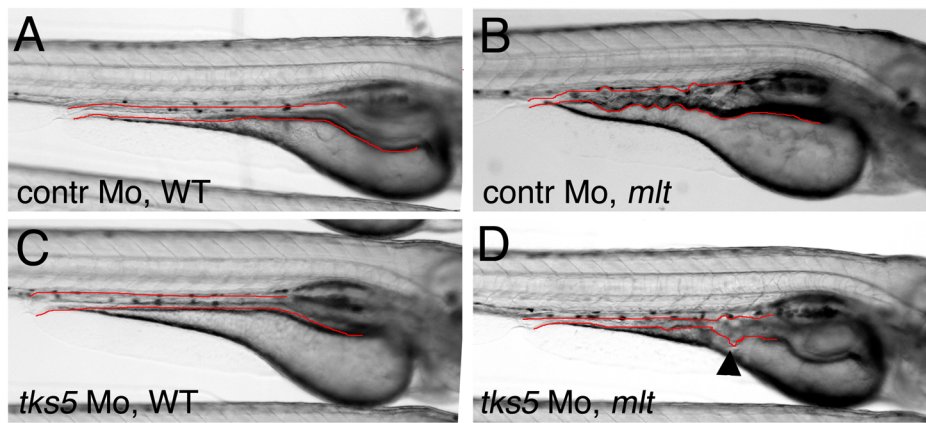

Figure S1

Supplement: Figure S1 — tks5 knockdown rescues epithelial invasion in the intestine of mlt mutant larvae. (A–C) Lateral images of live 5 dpf larvae. Control morpholino injected mlt larvae show cystic expansion of the intestinal epithelium (intestinal epithelium outlined in red; lateral view) (B), while only a small number of cysts can still be detected in the tks5 morpholino injected mlt larvae (D, arrowhead). The majority of the posterior intestine in these larvae resembles WT (A, C). Findings confirmed histologically (not shown; n = 6 mlt larvae). (PDF) [file pbio.1001386.s001.pdf]

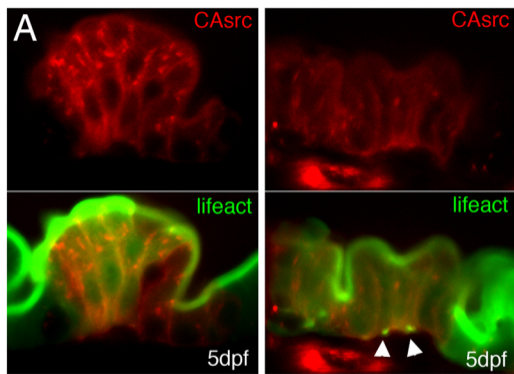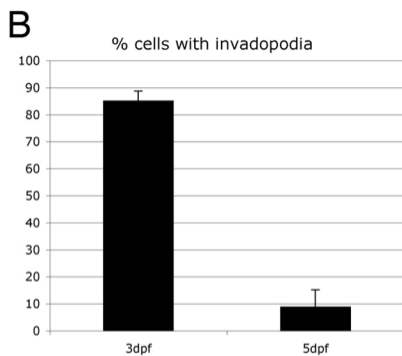

Figure S2

Supplement: Figure S2 — Src induces less invadopodia-like protrusions in mature intestinal epithelial cells than in epithelial progenitor cells. (A) Sagittal confocal scans through the intestine of a 5 dpf Tg(miR194:Lifeact-GFP) with mosaic expression of a caSrc-mCherry transgene. Only a small number of invadopodia-like protrusions are detected (green; arrowheads). (B) Quantification showed that invadopodia-like protrusions were detected in 89% of caSRC-mCherry positive cells in 3 dpf larvae (n = 28 cells in 5 larvae), while they were present in only 9% of cells in 5 dpf larvae (n = 64 cells in 5 larvae). Error bars, standard deviation. (PDF) [file pbio.1001386.s002.pdf]

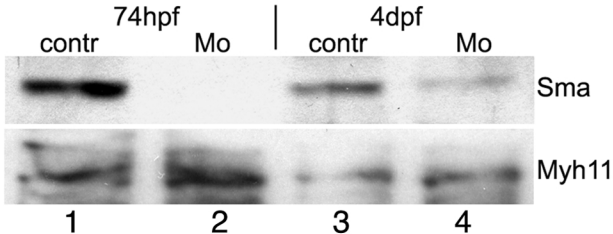

Figure S3

Supplement: Figure S3 — Smooth muscle actin (sma) knockdown rescues mlt mutants. (A–C) Western blot showing reduced Sma protein in the intestine of 74 hpf and 4 dpf Sma MO injected larvae (lane 2, 4) compared to a control MO (lane 1, 3). Myh11 protein levels are unaffected by the Sma knockdown (lanes 1–4). Each lane contains protein extracted from the intestines dissected from 30 larvae. This blot is representative of two independent experiments. (PDF) [file pbio.1001386.s003.pdf]

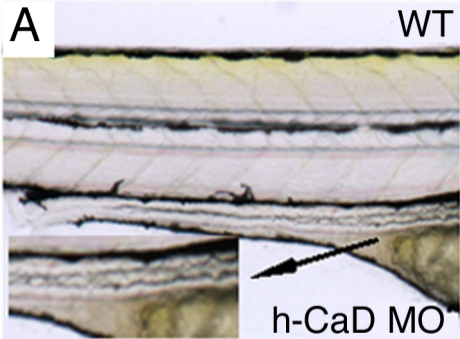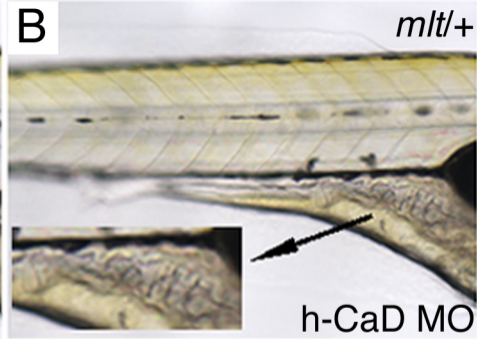

Figure S4

Supplement: Figure S4 — h-CaD knockdown triggers invasion in mlt heterozygotes. Lateral views of 5 dpf WT (A) and mlt/+ (B) larvae that had been injected with a splice-blocking morpholino (MO) that specifically targets h-CaD. Intestinal morphology is normal in the WT larva, whereas there is cystic expansion of the mlt/+ intestine. Histological analyses confirmed invasive expansion of the mlt/+ intestine (Figure 5D and 5E). Invasion was detected in 69% larvae from a mlt/+ intercross (n = 240) of which 66% were predicted to be mlt/+. 12 of 12 genotyped larvae were mlt/+. (PDF) [file pbio.1001386.s004.pdf]

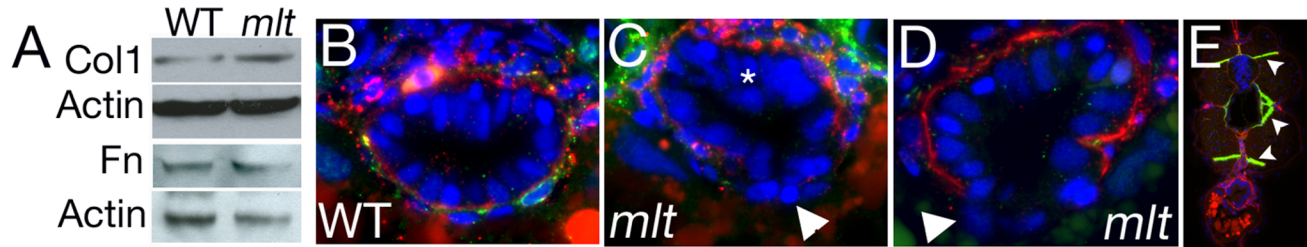

Figure S5

Supplement: Figure S5 — Invasive remodeling occurs without changes in matrix composition or FAK activation. (A) Western blot showing normal levels of Type 1 Collagen (Col1) and Fibronectin (Fn) in the intestine of 74 hpf mlt larvae compared with WT. beta-Actin serves as loading control. (B–D) Histological sections show no activation of FAK by phosphorylation (p-FAK, green) in the epithelium of WT or mlt larvae at 74 hpf. Basement membrane detected with anti-laminin staining (red) (asterisk, stratification; arrowheads, invasive sites). (E) Lower power image showing expected p-FAK (green) in the myoseptum of a WT larva. (PDF) [file pbio.1001386.s005.pdf]

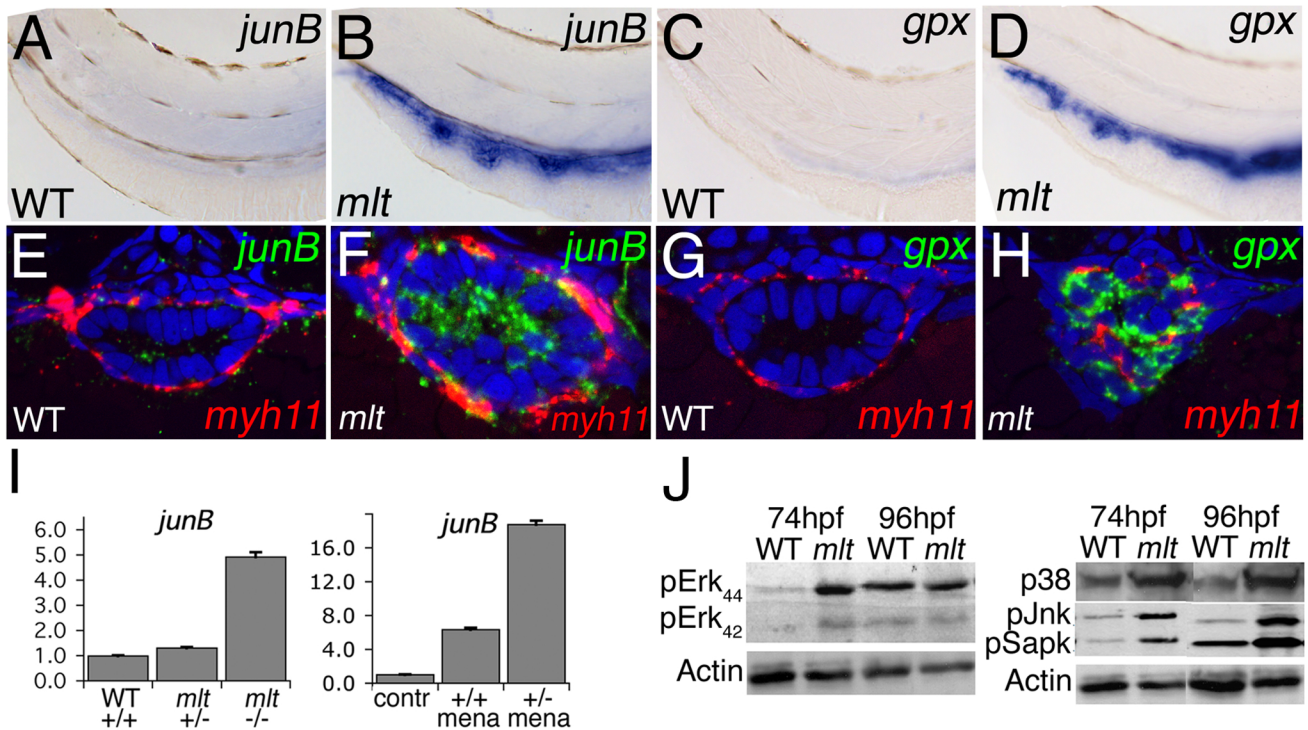

Figure S6

Supplement: Figure S6 — AP-1 transcription factors, ROS responsive genes, and MAP-Kinase signaling are activated in the mlt intestine. (A–D) Whole mount in situ hybridization shows strong expression (blue) of the AP-1 gene junB and the ROS activated gene gluthatione peroxidase (gpx) in the intestine of 74 hpf mlt (B, D) but not WT (A, C) larvae. n = 15 mlt and wild type larvae examined. (E, F) Histological cross-sections of whole mount specimens processed for fluorescent RNA in situ hybridization show strong junB expression (green) in mlt intestinal epithelial cells with only low level expression in smooth muscle cells (labeled red by myh11 expression). (G, H) Similarly, gpx expression (green) can only be detected in the mlt intestinal epithelium. n = 12 mlt and 12 wild type larvae examined. (I) Quantitative RT-PCR shows increased junB expression in the intestine of mlt homozygotes. junB expression is also increased in menadione treated wild type larvae (+/+), and to a greater degree in mlt heterozygous larvae (versus untreated wild type; contr). (J) Western blot showing phosphorylation of several components of the Map-Kinase signaling pathway in intestines dissected from mlt larvae at 74 hpf and 96 hpf. ERK is prematurely activated at 74 hpf in mlt. p38 (Mapk), Jnk, and Sapk are strongly activated in mlt but not WT larvae at 74 hpf and 96 hpf. beta-Actin, loading control. Western blots are representative of between two and four independent experiments. (PDF) [file pbio.1001386.s006.pdf]

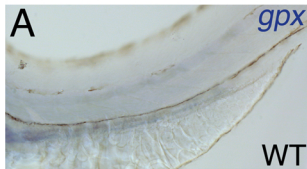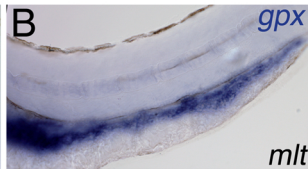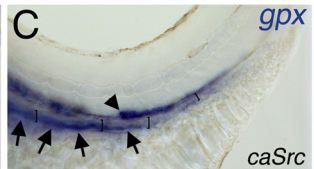

Figure S7

Supplement: Figure S7 — Comparable induction of gpx expression in WT larvae that express caSrc and homozygous mlt larvae. (A–C) Whole mount RNA in situ hybridization for gluthatione peroxidase expression (gpx, blue). gpx expression is low in the WT intestine (A), whereas it is markedly elevated in the mlt intestine (B; see also Figures 6C, 6D, S9G, and S9H). n = 15 mlt and 15 wild type larvae. (C) Mosaic expression of a caSrc transgene induces gpx expression in the wild type intestine (C, brackets and arrows). Interestingly, expression of caSrc in the pronephric duct (via the miR194 promoter) also induces gpx expression (C, arrowhead). n = 12 larvae. (PDF) [file pbio.1001386.s007.pdf]
